# Application of Multi-Strategy Controlled Rime Algorithm in Path Planning for Delivery Robots

**DOI:** 10.3390/biomimetics10070476

**Published:** 2025-07-19

**Authors:** Haokai Lv, Qian Qian, Jiawen Pan, Miao Song, Yong Feng, Yingna Li

**Affiliations:** 1 School of Information Engineering and Automation, Kunming University of Science and Technology, Kunming 650500, China; 20232104128@stu.kust.edu.cn (H.L.); cau_panjiawen@cau.edu.cn (J.P.); 20110050@kust.edu.cn (Y.F.); 12309147@kust.edu.cn (Y.L.); 2 School of Information and Engineering, Shanghai Maritime University, Shanghai 201306, China; miaosong@shmtu.edu.cn

**Keywords:** delivery robot, RIME optimization algorithm, fuch chaos, controlled elite, adaptive search, cosine annealing, path planning

## Abstract

As a core component of automated logistics systems, delivery robots hold significant application value in the field of unmanned delivery. This research addresses the robot path planning problem, aiming to enhance delivery efficiency and reduce operational costs through systematic improvements to the RIME optimization algorithm. Through in-depth analysis, we identified several major drawbacks in the standard RIME algorithm for path planning: insufficient global exploration capability in the initial stages, a lack of diversity in the hard RIME search mechanism, and oscillatory phenomena in soft RIME step size adjustment. These issues often lead to undesirable phenomena in path planning, such as local optima traps, path redundancy, or unsmooth trajectories. To address these limitations, this study proposes the Multi-Strategy Controlled Rime Algorithm (MSRIME), whose innovation primarily manifests in three aspects: first, it constructs a multi-strategy collaborative optimization framework, utilizing an infinite folding Fuch chaotic map for intelligent population initialization to significantly enhance the diversity of solutions; second, it designs a cooperative mechanism between a controlled elite strategy and an adaptive search strategy that, through a dynamic control factor, autonomously adjusts the strategy activation probability and adaptation rate, expanding the search space while ensuring algorithmic convergence efficiency; and finally, it introduces a cosine annealing strategy to improve the step size adjustment mechanism, reducing parameter sensitivity and effectively preventing path distortions caused by abrupt step size changes. During the algorithm validation phase, comparative tests were conducted between two groups of algorithms, demonstrating their significant advantages in optimization capability, convergence speed, and stability. Further experimental analysis confirmed that the algorithm’s multi-strategy framework effectively suppresses the impact of coordinate and dimensional differences on path quality during iteration, making it more suitable for delivery robot path planning scenarios. Ultimately, path planning experimental results across various Building Coverage Rate (BCR) maps and diverse application scenarios show that MSRIME exhibits superior performance in key indicators such as path length, running time, and smoothness, providing novel technical insights and practical solutions for the interdisciplinary research between intelligent logistics and computer science.

## 1. Introduction

The initial development of mobile robot technology was driven by the demands of high-risk industries such as industrial and military applications. These fields require extensive human resources while also facing challenges related to safety, complexity, and high precision. In the industrial sector, early robots were primarily used in nuclear industries, petrochemical plants, offshore operations, construction, outdoor applications (such as forestry and anti-personnel mine clearance), mining, and even recreational activities [1]. Over the past few decades, Finland’s VTT Technical Research Centre has been continuously researching mobile robot technology [2], successfully applying it to underground mines, electronics factories, and other diverse scenarios. In the military sector, the United States has developed mobile systems such as the Mobile Detection Assessment Response System (MDARS) and the Spiral Track Autonomous Robot (STAR) [3], significantly reducing safety risks in high-risk military operations. While these early mobile robots represented breakthroughs in functionality and operation, their applications remained largely confined to specific industrial and military environments.

With rapid advancements in internet technology, sensor technology, computer vision, and artificial intelligence, robots have gradually transitioned from fixed, controlled environments to more complex and dynamic applications. Mobile robots now play a crucial role in search and rescue [4], goods delivery [5], unmanned services [6], and geological exploration [7]. Particularly in the context of seasonal influenza outbreaks, demand for contactless delivery services has surged. Delivery robots, capable of efficiently performing contactless deliveries in diverse environments, have gained increasing public attention. However, a core challenge in their practical application lies in efficiently planning paths, avoiding obstacles, and minimizing energy consumption in complex environments. These challenges make path planning a key research focus.

The path planning problem for delivery robots is highly complex and dynamic, with the primary objective of generating an optimal or near-optimal path from the starting point to the destination within a given environment. The algorithm design must meet the following core requirements: first, the algorithm must possess global optimality, ensuring that it can find the shortest or safest path from the starting point to the destination on a large-scale map; second, the algorithm should minimize movement-related costs to the greatest extent possible, including path length, energy consumption, and time factors [8]. Traditional path planning algorithms are often limited by local optima, low computational efficiency, and poor environmental adaptability, making it difficult to meet the aforementioned requirements.

In recent years, bio-inspired optimization algorithms have demonstrated significant advantages in path planning due to their unique biologically inspired mechanisms. These algorithms, which simulate natural biological behaviors or physical phenomena (such as swarm intelligence, biological evolution, and physicochemical processes), provide novel approaches for solving path optimization problems in complex environments. For instance, Miao et al. [9] proposed an adaptive ant colony algorithm (IAACO), achieving comprehensive global optimization for robot path planning, while Wang et al. [10] introduced a novel flamingo path-planning algorithm, both demonstrating excellent performance in path optimization. However, according to the “No Free Lunch” theorem [11], no single optimization algorithm can perform optimally across all types of path-planning problems. Therefore, achieving a balance between algorithmic specialization and adaptability is crucial to ensuring search efficiency while enabling broad application. Researchers have improved various optimization algorithms, such as enhanced genetic algorithms [12], improved sparrow search algorithms [13], and enhanced dung beetle optimizer [14]. These advancements have helped overcome the limitations of traditional algorithms and have demonstrated significant potential in robot path planning. Consequently, researchers are increasingly focusing on bio-inspired optimization algorithms, particularly enhanced versions, for path planning applications.

The Rime Optimization Algorithm (RIME) [15] is a novel bio-inspired optimization algorithm inspired by the physical process of RIME formation in nature. By simulating the layered growth pattern of soft RIME on object surfaces and the penetrating crystallization characteristics of hard RIME crystals, it establishes a comprehensive “growth-penetration” collaborative optimization framework. This algorithm innovatively constructs a biomimetic mapping between meteorological phenomena and intelligent computation, offering advantages such as strong robustness, few parameters, and easy implementation. Compared to other bio-inspired algorithms such as the artificial bee colony algorithm [16] and the moth-flame optimization algorithm [17], RIME exhibits superior robustness and has been widely applied in engineering and mechanical fields. For example, Ismaeel et al. [18] applied RIME to parameter identification for proton exchange membrane fuel cells (PEMFC), optimizing fuel cell performance prediction models. Similarly, Abdel-Salam et al. [19] proposed an adaptive mutual-benefit chaotic RIME optimization algorithm, which significantly improved classification accuracy and reduced feature dimensions. Notably, RIME features a simple overall structure, and as demonstrated in [15], this emerging metaheuristic algorithm significantly outperforms most optimization algorithms in global search capability. Additionally, the overall time complexity of the RIME algorithm is O (*N_p_* × *D* × *T*) (where the main factors are the population size *N_p_*, problem dimension *D*, and the number of iterations *T*), indicating high computational efficiency. Based on these characteristics, the RIME algorithm can essentially meet the two core requirements of the aforementioned path planning problem.

However, directly applying the RIME algorithm to path planning for delivery robots still has some shortcomings. First, the soft frost search strategy is selected based on the adhesion coefficient *E*. Since the *E* value is relatively low in the early stages of the algorithm, the execution probability of soft frost search is low, resulting in most individuals being unable to update, which reduces the convergence speed and search capability of the algorithm. Second, the hard frost penetration mechanism directly updates individuals to the optimal position with a single update method, limiting the exploration ability of hard frost individuals and leading to insufficient population diversity. In the path planning process, insufficient early-stage search capability and lack of population diversity prevent the algorithm from fully exploring the search space, causing it to miss better path segments, generate overly long paths, and ultimately increase the robot’s travel time and energy consumption. Finally, the environmental factor in soft frost search decreases in a stepwise manner, leading to unstable variations in search step size. This causes significant differences in step values at different iteration stages, resulting in discontinuous changes in node coordinates. Consequently, the front-to-back coordinate differences and adjacent dimension differences increase, leading to a higher number of turning points in the path, ultimately reducing the smoothness of the generated path.

To address the aforementioned issues and further enhance the application potential of the RIME algorithm in delivery robot path planning, this paper proposes a novel Multi-Strategy Controlled RIME Optimization Algorithm (MSRIME). First, based on an analysis of various chaotic mapping techniques, MSRIME introduces a population initialization method using Fuch chaotic mapping [20], leveraging its chaotic properties to generate high-quality initial populations. Second, a controlled elite strategy and an adaptive search strategy are proposed to enhance the algorithm’s early-stage search capability and optimize the hard frost piercing mechanism, thereby improving population diversity and convergence speed, and effectively enhancing the global optimality of path planning. Additionally, a cosine annealing strategy is employed to replace the original stepwise step-size variation mechanism, ensuring smoother step-size adjustments in soft frost search, reducing the need for manual parameter tuning, and effectively avoiding local optima caused by abrupt step-size changes. By introducing a control factor *a*, multi-strategy synergy is achieved, further improving the overall performance of the algorithm. To validate the optimization performance of MSRIME, extensive comparative experiments and statistical analyses were conducted. Experimental results on the CEC2017 and CEC2022 benchmark test function sets demonstrate that MSRIME significantly outperforms comparison algorithms in terms of optimization capability, convergence speed, and stability. Finally, MSRIME was applied to path planning experiments for delivery robots in four scenarios with different Building Coverage Ratio (BCR). The results show that MSRIME outperforms comparison algorithms in key metrics such as path length, runtime, and path smoothness, further proving its superior performance in the field of path planning.

More significantly, as an enhanced version of RIME, MSRIME not only inherits RIME’s meteorological biomimetic characteristics but also integrates multiple optimization strategies through control factors, establishing a more universal “intelligence-nature” collaborative optimization framework. This not only offers a more efficient solution for delivery robot path planning but also fosters deeper interdisciplinary integration across biomimetics, computer science, and intelligent transportation.

## 2. Rime Optimization Algorithm (RIME)

RIME is an algorithm inspired by the natural formation of RIME frost. Rime occurs when water vapor in the air accumulates without condensing and then freezes at low temperatures, adhering to tree branches and other objects. The growth of RIME is influenced by factors such as temperature, wind speed, humidity, and air conditions, leading to variations in its formation under different circumstances. Additionally, due to environmental factors and growth limitations, RIME cannot grow indefinitely; once it reaches a relatively stable state, its growth ceases. Based on differences in environmental conditions and wind speed, RIME typically forms in two distinct types: soft RIME and hard RIME.

### 2.1. Initialization Phase

In the initialization phase of the RIME algorithm, the relevant parameters are set, including Population size (*N*), Dimensionality of the search space (dim), and Maximum number of iterations (*T*). The initial population *R*_*i*,*j*_, *I* = 1, 2, 3,…, *N*, *j* = 1, 2, 3,…, dim.

(1) $$R_{N\times \dim}=rand(N,\dim)\times (ub-lb)+lb$$

Here, *N* × dim represents the matrix space formed by the population size and dimensionality. *ub* denotes the upper bound of the feasible domain for the RIME population, while *lb* represents the lower bound. The initial population is randomly distributed within these boundaries to ensure diversity in the search space.

### 2.2. Soft Rime Search

**Figure 1:**
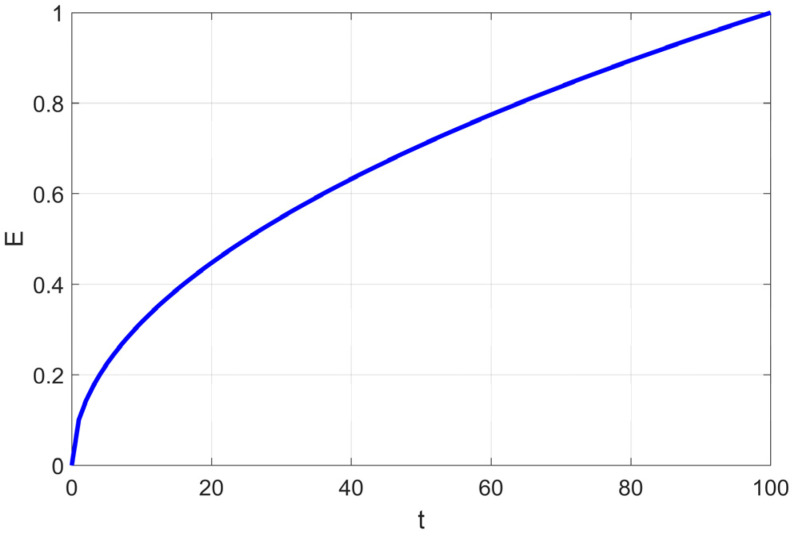
Variation Process of Coefficient *E*.

The exploration and exploitation phase of the RIME algorithm consists of soft RIME search and hard RIME piercing. The soft RIME search is primarily responsible for exploration and exploitation, while the hard RIME piercing accelerates the algorithm’s convergence. The details are as follows:

By simulating the movement of soft RIME agents within the RIME system, RIME introduces a soft RIME search strategy. This strategy is controlled by an attachment coefficient *E*, which regulates the condensation probability of soft rime. The value of *E* increases as the number of iterations progresses, following the variation process illustrated in Figure 1. To ensure a gradual and structured exploration-exploitation transition, the authors introduce two key elements: Environmental factor *β*: A trapezoidal function, where [] represents rounding operations, and *w* controls the number of segments in the trapezoidal structure. *β* decreases in a stepwise manner as iterations progress, simulating the effect of external environmental conditions. Directional control variable cos*θ*: Along with a random number *r*_1_, this variable determines the movement direction of agents in the population. Both *θ* and *β* change dynamically over iterations, allowing the algorithm to transition smoothly between large-scale exploration and fine-tuned exploitation.

**Figure 2:**
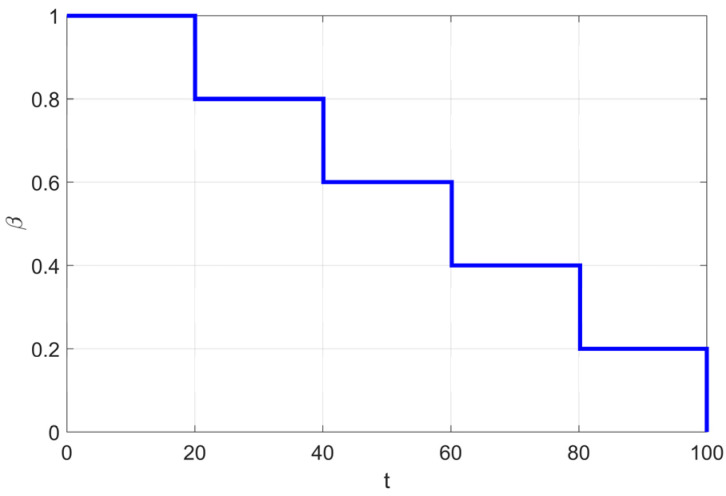
The process of change in the *β* of environmental factors.

This design ensures high efficiency and precision in the optimization process. The update formula for soft RIME search is given as follows:

(2) $$\theta =\pi \cdot \frac{t}{10\cdot T}$$

(3) $$\beta =1-[\frac{w\cdot t}{T}]/w$$

(4) $$E=\sqrt{\left(t/T\right)}$$

(5) $$R_{i,j}^{new}=R_{best,j}+r_{1}\cdot \cos\theta \cdot \beta \cdot (h\cdot (ub_{ij}-lb_{ij})+lb_{ij}),r_{2}\leq E$$

where *T* represents the maximum number of iterations and *t* is the current iteration number, controlling the progress of the algorithm. The upper and lower bounds for the j-th dimension of the *i*-th agent are denoted as *ub_ij_* and *lb_ij_*, respectively. *r*_2_ and *h* are random numbers within the range (0,1), where *r*_2_ determines whether the agent’s position is updated, and *h*, together with *ub_ij_* and *lb_ij_*, controls the position update range of the agent in each dimension. *β* is a trapezoidal decreasing function as shown in Figure 2, which works in coordination with other parameters to achieve the soft RIME search strategy.

### 2.3. Hard Rime Piercing

RIME introduces the hard RIME piercing mechanism by simulating the movement of hard RIME agents within the RIME system. The purpose of this mechanism is to ensure that all agents in the population have a certain probability of mutating into hard-rime agents, allowing them to continue the hard-rime exploration process and enhancing the algorithm’s convergence. The specific update formula is as follows:

(6) $$R_{i,j}^{new}=R_{best,j},r_{3}\leq F^{normr}(Si)$$

where *r*_3_ is a random number within the range (0,1), and *F^normr^*(*Si*) represents the normalized fitness value of the *i*-th agent. During the algorithm’s search process, Equations (5) and (6) jointly simulate the position update mechanism of the rime population.

### 2.4. Positive Greedy Selection Mechanism

The original algorithm employs a positive greedy selection mechanism, where, after each iteration, the updated fitness value of an agent is compared with its pre-update fitness value. If the updated fitness value is superior, both the agent and its fitness value are replaced accordingly. This ensures that the population evolves toward a better direction with each iteration, thereby enhancing exploration efficiency.

## 3. Multi-Strategy Controlled Rime Optimization Algorithm

### 3.1. Fuch Chaotic Mapping

In metaheuristic intelligent optimization algorithms, the quality of the initial population plays a crucial role in determining overall algorithm performance. A high-quality initial population enhances the global search capability of the algorithm and accelerates convergence, enabling faster identification of the optimal solution. Conversely, a low-quality initial population can restrict the search space, slow down the search process, and even cause the algorithm to fall into local optima, thereby diminishing its overall effectiveness.

However, in most swarm intelligence algorithms, agents in the initial population are generated randomly within a predefined range. This randomness introduces significant uncertainty in the initial population distribution. By replacing conventional random initialization with chaotic mapping, the chaotic properties can be leveraged to generate a highly diverse initial population, enhancing population diversity from the start. Several studies have demonstrated the effectiveness of chaotic mapping in improving initialization quality. Gao et al. [21] employed Tent chaotic mapping for initializing the whale optimization algorithm, increasing the diversity of the initial whale population, and ensuring a more uniform distribution of agent positions. Duan et al. [22] applied Tent chaotic functions along with a reverse learning mechanism to initialize the Grey Wolf Optimizer, resulting in more uniform and diverse population distributions. Wang et al. [23] used Henon chaotic mapping to initialize the Vulture Optimization Algorithm, significantly improving its search efficiency.

#### 3.1.1. Selection of Chaotic Mapping

The RIME algorithm employs a positive greedy selection mechanism during population updates. After each iteration, agents are updated based on their fitness values, retaining only the historical best value for each agent. Additionally, the hard RIME mechanism allows certain agents to directly update their positions to the current global best agent. Furthermore, the elite control strategy (Section 3.2) and the adaptive search equation (Section 3.3) are also designed around the optimal agent. As a result, both the original and improved versions of the RIME algorithm heavily depend on the best agent at each iteration. If the initial population is of low quality and the search process overly relies on the current best agent, the algorithm may only explore regions near a local optimum, increasing the risk of stagnation. To mitigate this issue, chaotic mapping can be used to generate a diverse, random, and unpredictable initial population, reducing the likelihood of the algorithm being trapped in local optima.

To further investigate the characteristics of chaotic mappings and determine the most suitable mapping method for MSRIME, this study conducted a systematic literature review of algorithm-related publications in four top-tier journals: IEEE Transactions on Pattern Analysis and Machine Intelligence, Computers in Industry, Applied Soft Computing, and Artificial Intelligence Review. Focusing on the period from 2004 to 2024, we used the search keywords “chaotic mapping method + optimization algorithm” and statistically analyzed the usage frequency of various chaotic mapping techniques. The results are shown in Table 1. The statistical data reveal that Logistic mapping and Tent mapping are the most frequently used, each appearing in over 200 studies over the past 20 years. The results demonstrate that both are widely applied in the enhancement of optimization algorithms. In addition, Multidimensional mappings enable direct interconnection of signals with similar characteristics, which facilitates effective processing and generation of data points in high-dimensional spaces, making them particularly suitable for high-density data scenarios [24]. Although the Fuch mapping appears less frequently in the statistical table, recent studies have revealed its distinctive advantages [25]: parameter-independent strong robustness, aperiodic chaotic stability, and exceptional harmonic spread-spectrum performance. These unique characteristics render it more practical than conventional chaotic mappings in specific application scenarios and technical domains. To ensure the comprehensiveness of this study, we selected not only highly cited one-dimensional chaotic maps (Logistic map and Tent map) but also incorporated a multi-dimensional chaotic map (Henon map) and a one-dimensional chaotic map with multiple unique properties (Fuch map) for more in-depth investigation.

#### 3.1.2. Initial Value Sensitivity Analysis

The aforementioned chaotic mappings share two crucial characteristics: initial value sensitivity and chaotic properties. Initial value sensitivity refers to the phenomenon in chaotic systems where minute changes in initial conditions can lead to significant differences in system trajectories. In chaotic mappings, higher initial value sensitivity indicates that the method can generate an initial population more likely to contain high-quality agents, thus accelerating algorithm convergence.

To assess the sensitivity of chaotic systems to initial values, the bit change rate can be employed as a quantitative metric. As noted in reference [20], when a system’s initial value undergoes a minor perturbation within the range of 10^−6^, observable changes occur in some bit values within the chaotic sequence. The bit change rate is calculated as the ratio of the number of changed bits (*b*) to the total number of bits (*B*), expressed as a percentage (*b*/*B* × 100%). A higher percentage indicates greater sensitivity of the system to initial conditions. In this study, we adopted the experimental protocol described in the reference to calculate the bit change rate for the selected chaotic maps. For the two-dimensional Hénon chaotic map, the initial values for both dimensions were set to the same numerical value. Table 2 presents the results of the initial value sensitivity analysis for four chaotic maps.

From the table, it is evident that the Fuch map exhibits the highest overall bit change rate, demonstrating the strongest sensitivity to initial values. The Tent map follows, while the Logistic map and Hénon map show relatively weaker sensitivity to initial conditions.

#### 3.1.3. Chaotic Property Analysis

The Lyapunov exponent is a key indicator for assessing the chaotic characteristics of a system [26]. A positive Lyapunov exponent indicates chaotic behavior, with larger values corresponding to more pronounced chaotic characteristics and higher degrees of chaos. This paper conducts a Lyapunov exponent analysis for four types of chaotic mappings. In chaotic mappings, *df*/*dx* characterizes the degree of trajectory separation, with 0 being the critical point for orbit separation in chaotic systems. For the initial state *x*_0_, the trajectory *x*_1_, *x*_2_, *x*_3_, … can be obtained through iterative chaotic mapping *f*(*x*), and a variable *k* is introduced to represent the initial infinitesimal distance between the two trajectories. Let *λ* denote the trajectory separation exponent during each chaotic iteration. The separation distance between trajectories after n iterations can be expressed as *f_n_*(*x*_0_ + *k*) − *f*(*x*_0_) = *k*e*^λ^*. Substituting this into the chaotic mapping relationship, and letting *k* approach an infinitesimal value and *n* approach infinity, the Lyapunov exponent *λ* values for the four types of chaotic mappings were calculated, with the results shown in Table 3.

(7) $$e^{\lambda }=\left|\frac{f_{n}(x_{0}+k)-f(x_{0})}{k}\right|=\underset{n\to \infty }{\lim}\left|\frac{d^{n}(x)}{d_{x}}\right|$$

Based on the comparison of Lyapunov exponents, the Fuch chaotic mapping exhibits the strongest chaotic characteristics, followed by the Tent chaotic mapping, while the Logistic and Henon mappings show weaker chaotic behavior.

Mappings with both high initial value sensitivity and high chaos degree are clearly more suitable for the RIME algorithm. Based on the above analysis, the Logistic and Henon mappings exhibit poor initial value sensitivity and chaotic characteristics, whereas the Fuch and Tent mappings satisfy the required conditions.

#### 3.1.4. Comparison of Chaotic Mappings

To scientifically evaluate the performance differences between Fuch mapping and Tent mapping in population initialization, this study designed a systematic comparative experimental scheme. Two variants of the RIME algorithm improved by chaotic mapping were selected: the FRIME algorithm using Fuch mapping and the TRIME algorithm using Tent mapping, while retaining the original RIME algorithm with random initialization as a control benchmark. The experiments selected the first 10 benchmark functions from the CEC2017 test set used by the original algorithm (including the F2 function, which has been officially removed but retained for comparison completeness) for performance evaluation. The population size was set to 50, the number of iterations to 100, and the dimension to 30, and each experiment was run 20 times independently.

Table 4 presents the statistical intervals of the optimization results for the three algorithms over 20 independent runs, where the interval endpoints represent the minimum (left endpoint) and maximum (right endpoint) values of the optimal solutions, respectively. To highlight the algorithmic performance advantages, the best minimum and maximum endpoint values for each test function are bolded. The experimental data show that the FRIME algorithm exhibits the highest frequency of bolded values across most test functions, demonstrating significant advantages. These results indicate that the initialization strategy based on Fuch mapping not only approximates the global optimal solution more stably but also achieves significantly higher solution accuracy than the compared algorithms.

The dispersion of the initial solutions often determines the optimization speed of the algorithm and the probability of getting trapped in a local optimum. From the above analysis, it can be seen that, compared to using Tent chaotic mapping and random generation methods, initializing the population with Fuch chaotic mapping results in a more orderly distribution, thereby minimizing the likelihood of the algorithm getting trapped in a local optimum. Therefore, this paper adopts the Fuch chaotic mapping to enhance the initialization method, with its mathematical model defined as follows:

(8) $$R_{x+1}=\cos(\frac{1}{R_{x}^{2}})\cdot (ub-lb)+lb$$

In the Fuch chaotic mapping, *R_x_* represents the chaotic variable, and *R_x_* ≠ 0; Equation (8) is used to generate a chaotic sequence. The initial value *R*_0_ is randomly selected from the range [0,1]. Through the iteration formula, a series of chaotic values *R*_1_, *R*_2_, …, *R_max_* are generated; *x* represents the iteration count; and *max* is the maximum iteration count. The specific process is as follows: in the population initialization stage of the MSRIME algorithm, the initial information is first obtained, including population size *N*, spatial dimension dim, maximum iteration count *T*, and the upper and lower bounds of the search space, *ub* and *lb*. The random number sequence is generated using Equation (8), and the random number sequence corresponding to the population agents is linearly mapped to its upper and lower bounds, resulting in the initial population *R*.

### 3.2. Control Elite Strategy

In the process of solving optimization problems, the dynamic evolution of the population is crucial for improving the overall performance of the algorithm. Reasonable population changes can not only enhance the algorithm’s global search ability but also significantly accelerate the convergence process, thereby finding the optimal solution in a shorter time. Although the frost-ice population initialization has been optimized using Fuch mapping, as discussed in Section 3.1, inherent flaws in RIME’s evolution strategy may still result in low-quality populations (analyzed in detail in the next section). To address this challenge, this section introduces a new search strategy: the control elite strategy.

During the iteration process of the original frost-ice algorithm, four types of frost-ice agents satisfying different conditions may appear:

(1) Agents that satisfy only the soft frost search condition but not the hard frost puncture condition, performing soft frost search.
(2) Agents that satisfy only the hard frost puncture condition but not the soft frost search condition, performing hard frost puncture.
(3) Agents that satisfy both the soft frost search and hard frost puncture conditions, performing hard frost puncture.
(4) Agents that satisfy neither the soft frost search nor the hard frost puncture conditions, performing no operations.

The conditions for generating the aforementioned four types of individuals have different values at various stages of the algorithm’s execution. This can lead to a series of defects and shortcomings in the population of individuals during the update process. To visually illustrate how the number of agents meeting different conditions changes during various iterative phases of the algorithm, this study conducted a 100-iteration experiment with 100 agents from the original algorithm.

**Figure 3:**
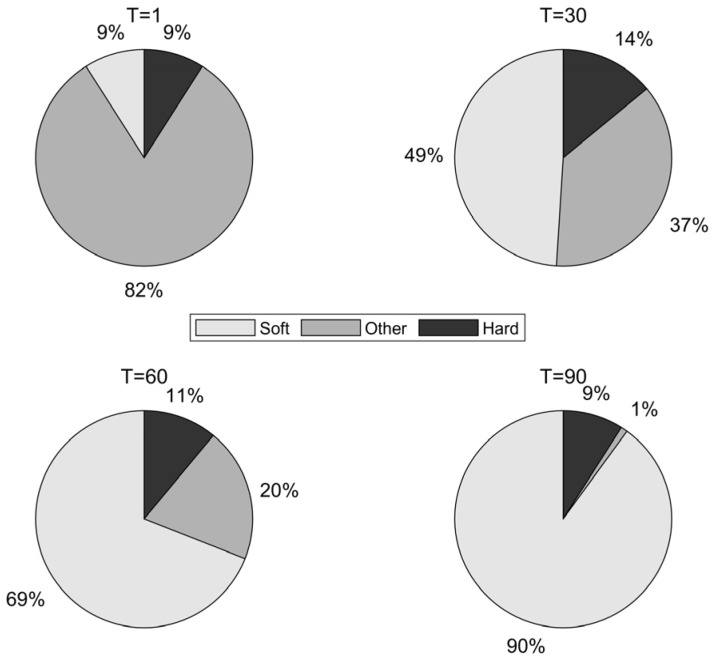
Population Distribution Diagram.

The data at iterations 1, 30, 60, and 90 were selected to simulate the early, early-middle, middle-late, and late stages of algorithm execution. The proportion of agents satisfying soft frost search (Condition 1), hard frost search (Conditions 2 and 3), and other agents (Condition 4) was calculated, and the results were visualized as the pie charts shown in Figure 3.

Since the adhesion coefficient E in the soft frost search is relatively low in the early stages (as shown in Figure 1), the probability of executing soft frost search is extremely low. Figure 3 also shows that in the early iterations, the proportions of the soft frost population (Soft) and hard frost population (Hard) are relatively low. Most agents remain stagnant in the initial phase due to satisfying Condition (4), resulting in low search efficiency during the early iterations.

In contrast, the proportion of agents satisfying the “Other” condition is relatively high in the early stages. Enhancing the search capability of these agents in the early phase can effectively improve the algorithm’s optimization performance. The core idea of the elite learning strategy is to leverage elite agents—those with high fitness in the current iteration—to guide the update of other agents, thereby accelerating convergence toward better solutions.

Specifically, this study applies the elite learning strategy to a subset of agents satisfying Condition (4), enabling some agents to move toward higher-quality agents. This defines a new Condition (5): If an agent satisfies Condition (4) and a randomly generated value is less than a predefined probability a, the agent executes the elite learning strategy.

During the iteration process, agents satisfying Condition (4) may fall into the following two scenarios:

Scenario 1: Some agents have relatively good fitness but satisfy Condition (4) due to a large random number. These agents can be retained.

Scenario 2: Some agents have poor fitness and also satisfy Condition (4) due to a small random number. These agents should execute the elite learning strategy.

**Figure 4:**
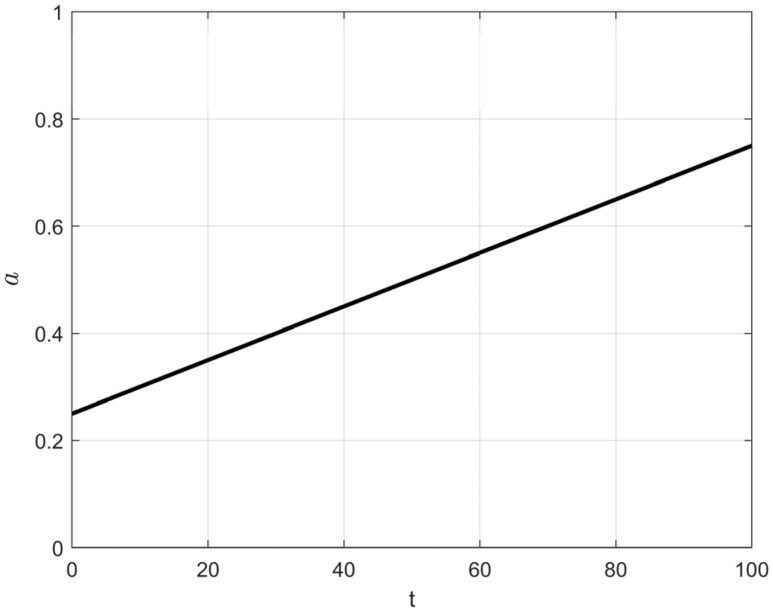
The variation process of the control factor.

To better distinguish between these two types of agents and to adapt to the increasing overall fitness of the frost population as the iteration process progresses, an adaptive control factor, *a*, is introduced to regulate the triggering probability of the elite learning strategy. The calculation of *a* is given by Equation (9), and its variation trend is shown in Figure 4. As seen in Figure 4, a increases linearly from an initial value of 0.25 to 0.75 as the number of iterations t increases. This linear increase controls the number of agents satisfying Condition (5) throughout the iterations. Furthermore, as shown in the elite learning formula (Equation (10)), the use of *a* along with the normalized fitness value of an agent as the triggering condition ensures that only agents with relatively small random numbers and low fitness (i.e., agents corresponding to Scenario 2 described earlier) execute the elite learning strategy.

(9) $$a=\frac{T/2+t}{2T}$$

The elite learning strategy formula is given as Equation (10):

(10) $$R_{i,j}^{new}=\frac{R_{i,j}+R_{best,i}}{2},F^{normr}(Si)\leq r_{3}<a,r_{2}\geq E$$

(11) $$F=\sum_{i=1}^{n}{\left(\sum_{j=1}^{i}x_{j}\right)}^{2}$$

**Figure 5:**
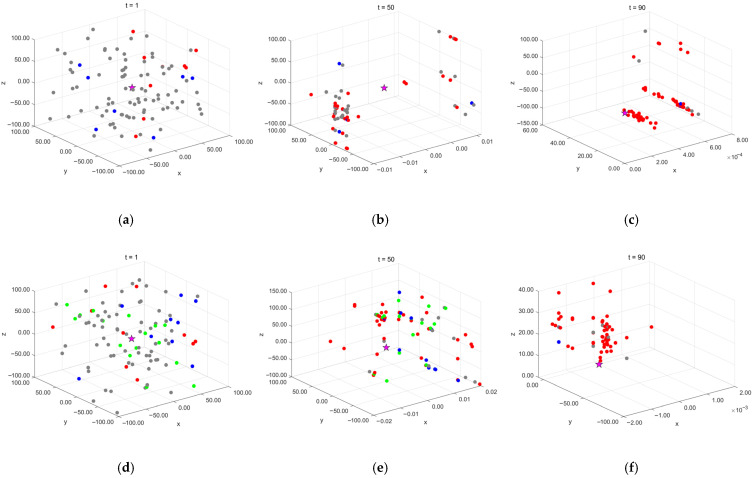
Evolution of the Controlled Elite Population. (**a**) Early-stage population without the controlled elite strategy. (**b**) Mid-stage population without the controlled elite strategy. (**c**) Late-stage population without the controlled elite strategy. (**d**) Early-stage population with the controlled elite strategy. (**e**) Mid-stage population with the controlled elite strategy. (**f**) Late-stage population with the controlled elite strategy.

To more intuitively investigate the impact of the controlled elite strategy on the dynamic evolution of the population, this study selects the classical optimization test function, Schwefel’s Problem 1.2 [27], as the experimental subject, as shown in Equation (11). This function has a well-defined global optimum, easily observable convergence behavior, a nonlinear optimization process, and strong coupling between variables. These characteristics effectively reflect an algorithm’s performance in complex search spaces, making it suitable for analyzing population dynamics and demonstrating algorithm performance. The initial population size is set to 100, with the algorithm iterating 100 times. Population distribution is sampled and analyzed at three key iteration points (initial state t = 1, mid-iteration t = 50, and late iteration t = 90), with results shown in Figure 5.

In the figure, pentagram nodes represent the theoretical optimal solution, red dots indicate soft frost population agents, blue dots represent hard frost population agents, gray dots denote non-mutated agents, and green dots signify newly introduced controlled elite population agents. The experimental results show that, without the controlled elite strategy, the algorithm in the early iteration phase is primarily dominated by non-mutated agents (gray dots), indicating limited search capability at this stage. However, after introducing the controlled elite strategy, some non-mutated agents in the early iterations are assigned elite search capabilities, transforming into elite population agents (green dots).

As the iterations progress, elite population agents maintain a certain proportion in the mid-iteration phase (t = 50) but significantly decrease in the late iteration phase (t = 90), demonstrating that the control factor effectively regulates the probability of elite agent generation. The introduction of the control factor ensures that the early iteration phase is still dominated by non-mutated agents, whereas the late iteration phase is primarily driven by the soft frost and hard frost populations. This mechanism enhances early-stage exploration while preventing premature convergence or insufficient exploitation due to excessive or premature elite population generation.

### 3.3. Adaptive Search Strategy

As shown in Figure 3, the proportion of individuals satisfying the Hard Frost Penetration (Hard) condition remains relatively stable, fluctuating between approximately 5% and 20% throughout the iteration process. However, as shown in Equation (6), the update mechanism of the Hard Frost strategy exhibits high uniformity, leading to a lack of the diversity required for population search and a reduced ability to explore local regions. To address this issue, this paper proposes an adaptive search strategy applied to the update process of the Hard population.

The adaptive search strategy refers to an optimization approach that improves the search process by learning and adapting during iterations, enabling the algorithm to explore and exploit information in the search space more effectively. This strategy dynamically adjusts the search direction, step size, or search space based on problem characteristics and feedback from the search process to enhance algorithm efficiency and performance. While the introduction of the elite learning strategy mitigates the weak search capability in the early and mid-iterations of the algorithm, the search range and accuracy of the population still require further optimization. Beyond individuals satisfying conditions (4) and (5), a portion of the population meets conditions (2) or (3) and follows the Hard Frost update mechanism during the early, middle, and late stages of the iteration process. Enhancing the search range of these individuals in the early and middle stages, as well as improving their local exploitation capability in the later stages, is also a key factor in effectively increasing algorithm efficiency.

In the original algorithm, individuals that satisfy conditions (2) or (3) are directly generated at the optimal individual location, which leads to a reduction in population diversity during the iteration process. To enable individuals that meet the hard frost search condition to explore a larger range around the global optimal position in the early and middle stages, and to conduct refined development near the global optimal position in the later stages, this section integrates the adaptive search strategy with the control factor *a* described in Equation (9) and proposes an adaptive search equation. This equation replaces the original hard frost mechanism (Equation (6)) and adjusts the search range of the hard frost process through the adaptive variation of the control factor *a*. Furthermore, during the iteration process, the value of 1 −*a* will not fall below 0.25, ensuring that even in the later stages of iteration, there is a small probability of generating relatively large step sizes, which increases the algorithm’s ability to escape local optima. The specific equation is as follows:

(12) $$R_{i,j}^{new}=R_{best,j}+(2r-1)(1-a)(R_{best,j}-R_{i,j}),r_{3}<F^{normr}(Si)$$

where *r* is a random number between 0 and 1, and *a* is the control factor. As the number of iterations increases, the search range of the hard frost mechanism will adaptively decrease.

Through the application of the elite strategy in Section 3.2, MSRIME introduces some elite-characterized Other individuals during the exploration process to enhance the algorithm’s search capability in the early and middle stages. At the same time, the adaptive search strategy optimizes the updating method for the Hard population, allowing it to adaptively increase the search range of the population individuals. The simultaneous application of these two strategies, working together in harmony, can effectively improve the overall search capability of the population.

### 3.4. Cosine Annealing Strategy

As mentioned in Section 2.2, *β* is a trapezoidal decreasing function (Equation (3)) used in the original algorithm to simulate changes in the external environment. Its value decreases with the number of iterations, enabling a stepwise transition between large-scale exploration and small-scale exploitation. The parameter *w* in Equation (3) needs to be manually adjusted to control the number of trapezoidal segments.

In reference [15], the parameter *w* is set to 5, and the step size function variation is plotted in Figure 2. As shown in the figure, the step size gradually decreases with the increase in the number of iterations. However, as indicated by the convergence curves (a) and (d) in Figure 11 of Section 5, when the number of iterations reaches 4/5 of the total iterations (i.e., at the transition point from the fourth to the fifth segment of the trapezoidal function), the RIME algorithm’s convergence curve has already stabilized near the current optimal solution. This phenomenon can be attributed to the five-segment trapezoidal function design, where the step size undergoes abrupt changes at certain critical points. Such discontinuous step size variations disrupt the search process, causing the algorithm to prematurely focus on local regions for fine-tuned searches before fully exploring the global solution space, resulting in missing the global optimal solution.

Additionally, the trapezoidal function follows a predefined fixed pattern, lacking the ability to dynamically adjust based on the current search state. Furthermore, in the process of RIME ice formation, the surrounding environment does not change in a stepwise manner. To make the environmental factor *β* better align with the physical variations of the external environment and to smoothly adjust the step size of Soft Frost agents, the cosine annealing strategy is introduced to replace the trapezoidal function *β*.

In deep learning, model training typically relies on gradient descent and its variants to iteratively adjust model parameters. The learning rate is a crucial hyperparameter in these algorithms, as it determines the step size for each parameter update. Cosine annealing is a widely used learning rate scheduling strategy applied to optimize the training process of deep learning models [28]. It is formulated as Equation (13), where *η_t_* represents the learning rate at time step t (i.e., the current iteration count), while *η_min_* and *η*_max_ denote the minimum and maximum learning rates, respectively, and *T* is the iteration period length (i.e., the maximum number of iterations).

(13) $$\eta_{t}=\eta_{\min}+\frac{1}{2}(\eta_{\max}-\eta_{\min})(1+\cos(\frac{t\pi }{T}))$$

Li et al. [29] found through research that, compared to the trapezoidal decay approach, cosine annealing is a superior step size adjustment method. Unlike the trapezoidal function, the smooth decay of cosine annealing facilitates a more stable convergence to better solutions during the iterative exploration process. This adjustment method helps reduce oscillations caused by abrupt step size changes, and it eliminates the need for manual parameter tuning by allowing automatic adjustments based on a predefined formula, thereby reducing the complexity of hyperparameter tuning. As demonstrated in the convergence curves presented in Section 5, in some test functions, MSRIME consistently escapes local optima in the later stages of iteration, outperforming the original algorithm.

**Figure 6:**
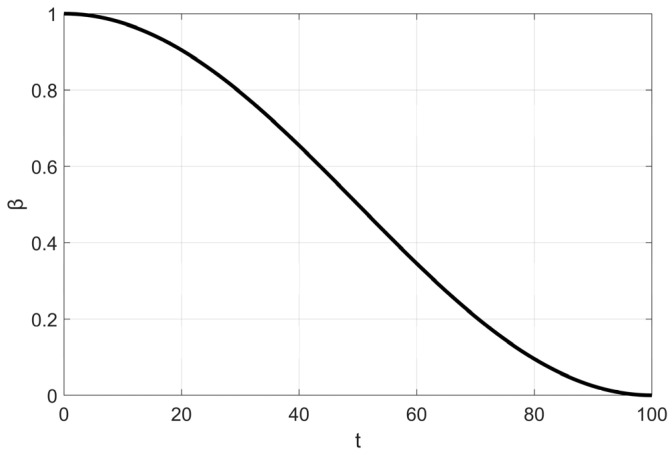
Cosine Annealing Image.

This observation confirms that the combination of cosine annealing and adaptive search strategies effectively expands the search range, thereby increasing the probability of escaping local optima. Consequently, this paper adopts cosine annealing as the environmental factor, with its variation range set to [0,1]. The variation trend is illustrated in Figure 6, and the final formulation is given as follows:

(14) $$\beta =\frac{1}{2}(1+\cos(\frac{t\pi }{T}))$$

### 3.5. Selection of Control Factors and Multi-Strategy Coordination Analysis

Section 3.2 elaborates on the mechanism for setting the control factor *a*, which serves as the trigger condition for the elite strategy. The value of *a* is required to increase monotonically. While functions other than Equation (9) (e.g., Equations (15) and (16)) also exhibit this monotonic increase, this study ultimately selected Equation (9) as the control factor.

**Figure 7:**
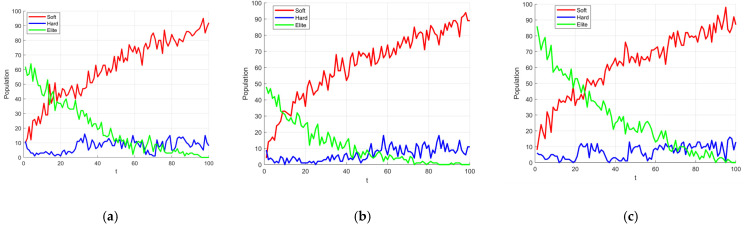
Parameter analysis experiment. (**a**) a1, (**b**) a2, (**c**) a3.

The primary objective of introducing the controlled elite strategy is to enhance the algorithm’s exploratory capability during the early stages of iteration, to a certain extent. However, it is crucial to prevent a large number of ‘frost’ individuals from abruptly transforming into ‘elite’ individuals, which could lead to premature convergence of the algorithm. Concurrently, a small proportion of elite individuals must continue to be generated and integrated into the exploitation process during the mid-to-late stages of iteration. To validate the appropriateness of this parameter selection, we conducted experiments on population size variation using Schwefel’s Problem 1.2 (from Section 3.2) as the test function. The population size was set to 100, and the number of iterations was 100. We compared three different parameter settings for *a*: Equation (9)’s *a* (hereafter referred to as a1), Equation (15)’s *a* (hereafter referred to as a2), and Equation (16)’s *a* (hereafter referred to as a3). The experimental results are presented in Figure 7.

(15) $$a=\frac{t}{2T}$$

(16) $$a=\frac{T+t}{2T}$$

As depicted in Figure 7, the green lines represent the population changes when the controlled elite strategy is triggered. However, it is evident that the curve in Figure 7b (using a2) essentially ceases to generate elite individuals after approximately 60 iterations. Conversely, the curve in Figure 7c (using a3) shows that elite individuals constitute nearly 80% of the population in the very early stages of iteration. Both of these behaviors contradict our initial design intention. Furthermore, the upper limit of the parameter a1 = 0.75 can complement the cosine annealing strategy, ensuring that ‘hard frost’ individuals maintain a sufficiently large step size under the adaptive search strategy (Section 3.3) during the later stages of iteration. This compensates for the reduced ability of ‘soft frost’ individuals to escape local optima under cosine annealing conditions.

To validate the selection of parameters and the effectiveness of the multi-strategy combination, this section utilizes Schwefel’s Problem 1.2 as the test function. We set the initial population size to 50, ran 100 iterations, and repeated the experiment 30 times. The results are presented in Table 5. In this table, RIME1 represents the RIME algorithm integrated with the Fuch chaotic map; RIMEa1, RIMEa2, and RIMEa3 represent the RIME algorithm using the elite control strategies with parameters a1, a2, and a3 respectively; RIME2 denotes the RIME algorithm with the adaptive search strategy; and RIME3 refers to the RIME algorithm incorporating the cosine annealing strategy.

**Figure 8:**
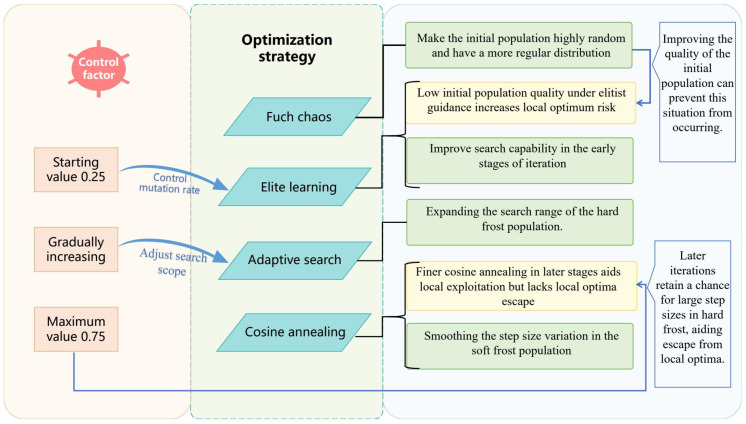
Framework of Multi-Strategy Coordination and Integration.

The experimental results show that among the RIMEa1, RIMEa2, and RIMEa3 groups, RIMEa1 performed best in terms of both average value (AVG) and standard deviation (STD), further confirming the rationality of selecting parameter a1 for the control factor. Conversely, RIMEa3 exhibited the poorest optimization performance, indicating that inappropriate parameter settings can lead to an excessive generation of elite individuals in the early stages, causing premature convergence. Notably, the MSRIME algorithm, which integrates a multi-strategy collaborative mechanism, significantly outperformed all single-strategy improved algorithm variants in convergence performance. Figure 8, which analyzes the multi-strategy complementary mechanism, shows that MSRIME demonstrates significant advantages in both optimization accuracy and stability compared to algorithms solely employing the Fuch chaotic map (RIME1), the elite control strategy (RIMEa1), or the cosine annealing strategy (RIME3). This outcome confirms that a carefully designed multi-strategy collaborative framework can not only effectively reduce the complexity of parameter tuning but also fully leverage the complementary strengths of each strategy, thereby comprehensively enhancing the algorithm’s overall optimization performance.

### 3.6. Time Complexity Analysis

Reference [30] indicates that the time complexity of the original RIME algorithm is primarily determined by the population initialization and update operations (including soft frost search and hard frost search). Its overall time complexity can be expressed as *O* (*N_p_* × *D* × *T*).

Compared to the original RIME algorithm, the proposed MSRIME algorithm introduces improvements in the following aspects: First, the population initialization method is replaced from random generation to Fuch chaotic mapping, but the time complexity of population initialization remains O (*N_p_* × *D*), consistent with the original method. Second, in the update operations, the hard frost search strategy is improved into an adaptive form, while its time complexity remains unchanged. Additionally, a cosine annealing strategy is introduced to replace the step-size adjustment mechanism in the original soft frost search, and the time complexity of the cosine annealing strategy is *O* (*T*), the same as the original step-size adjustment method. Finally, the elite control strategy is introduced during the update process, and its control factor update has a time complexity of *O* (*T*). In summary, the overall time complexity of the update operations in the MSRIME algorithm remains *O* (*N_p_* × *D* × *T*), consistent with the complexity order of the original RIME algorithm.

### 3.7. Implementation of MSRIME

Based on the above analysis, this section presents the pseudocode implementation of the MSRIME algorithm (Algorithm 1). The algorithm begins by initializing the population using the Fuch chaotic map, which is known for its high sensitivity to initial values, thereby ensuring a diverse distribution of initial solutions within the search space. Subsequently, it proceeds into an iterative optimization process. At the start of each iteration, key control parameters (including *E*, *a*, etc.) and necessary random numbers are dynamically generated. Based on predefined conditions, the current population is adaptively divided into three sub-populations: the soft frost population is updated using Equation (5), the hard frost population using Equation (12), and the elite population using Equation (10). After each iteration, a strict positive greedy selection strategy is executed. Finally, upon reaching the predetermined maximum number of iterations, the algorithm outputs the optimal solution.
**Algorithm 1**: MSRIME Algorithm1Initialize the population using the Fuch chaotic mapping.2Obtain the current best agent and best fitness value.3While *t* ≤ *T*4  Generate random numbers $r_{2}$, $r_{3}$, update *E*, *a*, and *β* using Equations (4), (9), and (14).5If *r*_2_ < *E*6  Perform soft frost search using Equation (5).7End If8If *r*_3_ < Normalize fitness of *Si*9  Perform hard frost search using Equation (12).10Else If Normalize fitness of *Si* ≤ *r*_3_< *a && r*_2_
*≥ E*11  Execute the controlled elite strategy using Equation (10).12End If13If $F(R_{i}^{new})<F(R_{i})$
14  Select the optimal solution and replace it using a positive greedy selection mechanism.15End If16  *t* = *t* + 117End While

## 4. Qualitative Analysis of MSRIME

Qualitative analysis systematically explores and evaluates the properties, behavior, and characteristics of an algorithm through intuitive understanding, theoretical derivation, and experimental observation. In the study of optimization algorithms, qualitative analysis typically focuses on the overall behavior, performance trends, and performance under different conditions.

To this end, this section selects functions from the CEC2022 test function set (as shown in Table 5), which includes unimodal functions (f1), basic functions (f2–f5), hybrid functions (f6–f8), and composition functions (f9–f12). These functions are derived from corresponding basic test functions through translation, rotation, scaling, and composition, thereby increasing their complexity. This approach eliminates the defect of the basic test functions having the origin as the optimal point and removes the symmetry present in the solution space. As a result, these functions can comprehensively test the global search capability, local search capability, robustness, and adaptability of optimization algorithms.

The experiment is conducted with a problem dimension of 20, a population size of 50, and 500 iterations. The qualitative analysis of MSRIME is performed from two perspectives: First, by analyzing its convergence behavior, the convergence performance and search behavior of MSRIME are demonstrated. Then, the population diversity is explored to examine whether the algorithm can maintain a good diversity of solutions during the search process, thereby avoiding premature convergence.

### 4.1. Convergence Behavior Analysis

**Figure 9:**
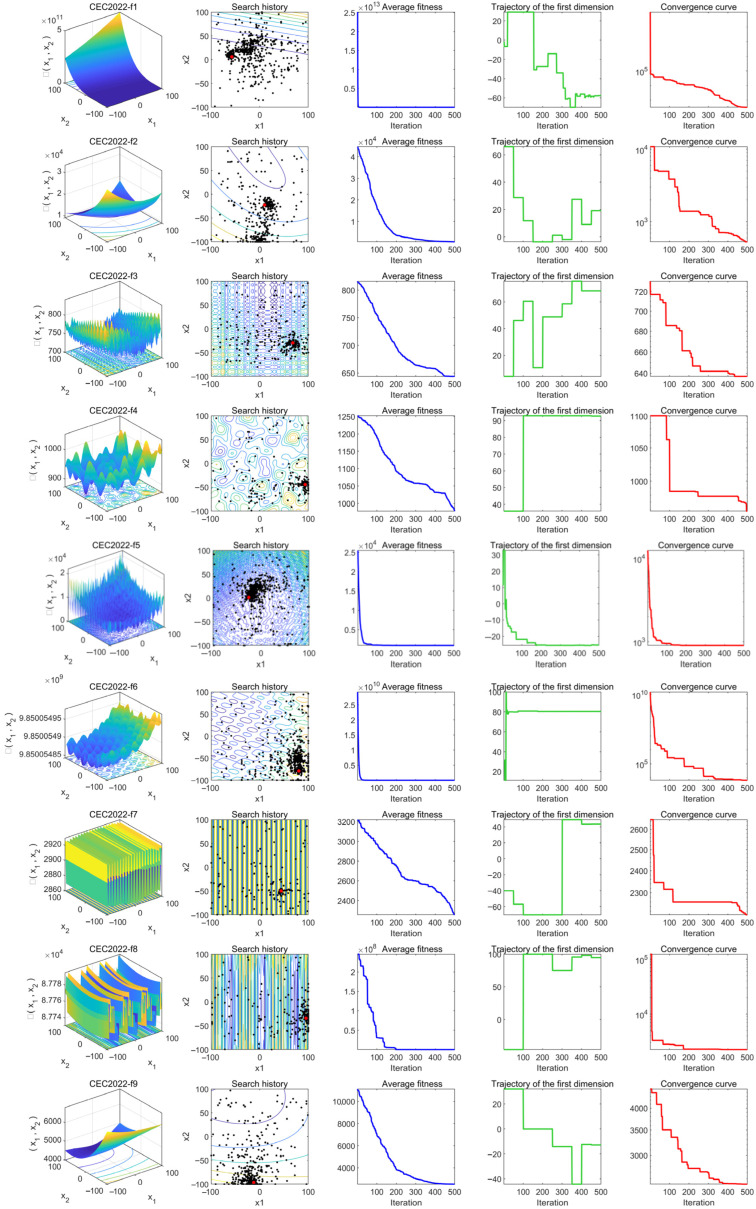

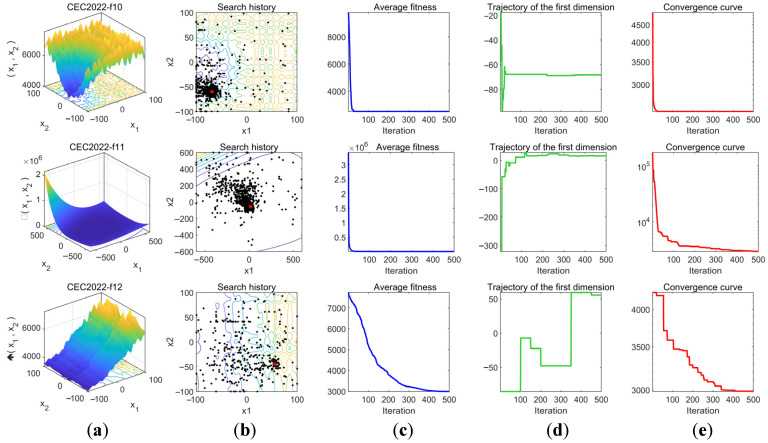
Convergence behavior analysis: (**a**) shows the functional characteristic diagram, (**b**) displays the search trajectory, (**c**) presents the variation trend of average fitness values, (**d**) illustrates the variation trend along the first dimension, and (**e**) demonstrates the changes in the optimal solution with iteration count.

The experimental results of the convergence behavior analysis are shown in Figure 9. To better illustrate the distribution characteristics of the test functions, column (a) in the figure presents the 3D shape of the objective functions, allowing readers to intuitively understand their properties. Column (b) records the search trajectories of the algorithm in the search space, where black dots represent the population distribution and red dots denote the optimal solution, reflecting the exploration range and movement path of the algorithm. Column (c) depicts the variation trend of the average fitness value, serving as a typical convergence indicator that clearly demonstrates the convergence trend of the algorithm. Column (d) tracks the changes in the population along the first dimension during optimization, providing insight into whether the population exhibits wide-range exploration, local search behavior, and convergence tendencies. Column (e) illustrates the change in the best-found objective function value over iterations, directly reflecting the convergence performance of the algorithm.

From column (b), it can be observed that across all functions f1-f12, the population trajectories of the MSRIME algorithm converge toward the optimal solution region, indicating that the search paths are relatively concentrated and the population effectively focuses on the target area, demonstrating strong convergence characteristics. In column (c), the overall fitness value curve rapidly declines and gradually stabilizes, indicating that the population fitness values improve quickly in the early stages and then gradually converge. Notably, in functions f1, f2, f5, f6, f8, f9, f11, f12, the fitness curves experience a sharp decline within the first one-third of the iterations. This suggests that the introduction of the controlled elite strategy significantly enhances the algorithm’s early exploration ability and accelerates convergence. Additionally, for functions f3, f4, and f7, the fitness curves exhibit another substantial decline even in the later iterations, demonstrating that the adaptive search and cosine annealing strategies effectively prevent the algorithm from being trapped in local optima. The one-dimensional trajectory in column (d) reveals that in most functions, the curves exhibit noticeable jumps throughout the process. This continuous jump behavior indicates that the algorithm not only maintains a broad search space but also balances global exploration and local exploitation effectively. Finally, column (e) shows the trend of the best objective function value over iterations. In all functions, the descending convergence curves indicate that the algorithm progressively finds better solutions. Notably, in functions f1, f4, f6, and f7, the algorithm escapes local optima in the later iterations to discover better solutions. For functions f5 and f8, the optimal solution is found early in the mid-iterations. These observations further confirm the beneficial impact of the multi-strategy improvements on the algorithm’s convergence behavior.

### 4.2. Population Diversity Analysis

Population diversity is a critical metric for evaluating the range and uniformity of individual distributions, directly influencing an algorithm’s global search capability and convergence performance. This section analyzes the diversity variation trends at different iterative stages through population distribution visualization and diversity measurement curves.

**Figure 10:**
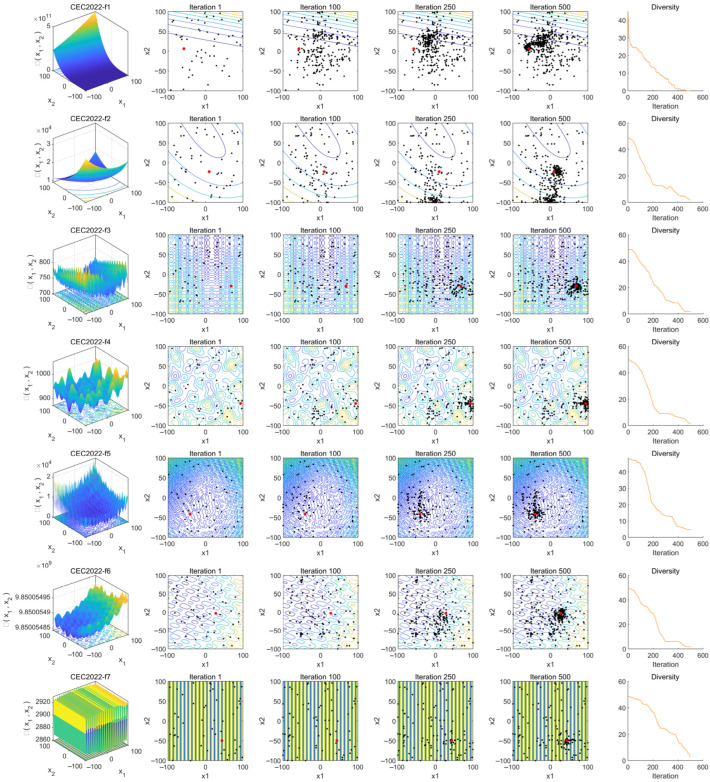

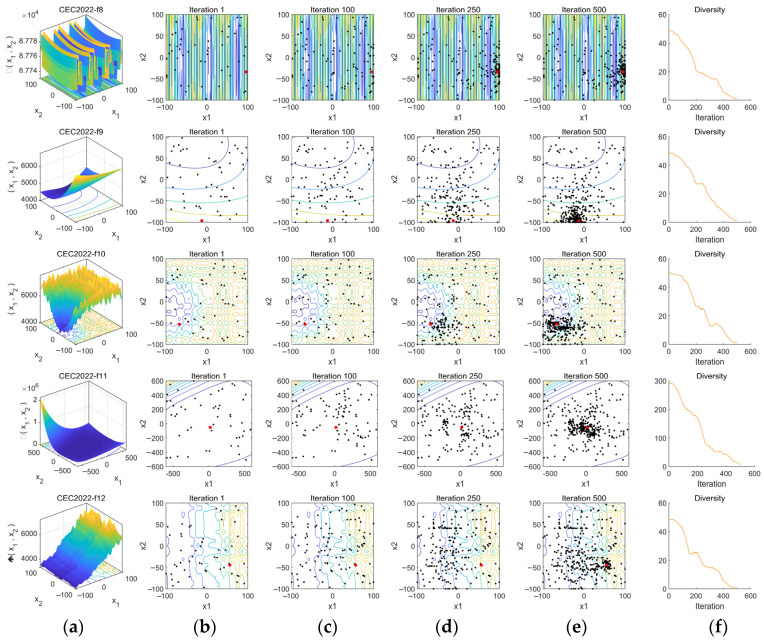
Population Diversity Analysis: (**a**) displays the function characteristic diagram, (**b**) shows the search trajectory after 1 iteration, (**c**) presents the search trajectory after 100 iterations, (**d**) illustrates the search trajectory after 250 iterations, (**e**) demonstrates the search trajectory after 500 iterations, and (**f**) provides the population diversity analysis.

In Figure 10, column (a) presents the characteristics of the target convergence functions. Columns (b) to (e) illustrate the search distributions of the optimization algorithm at different iteration stages (1st, 100th, 250th, and 500th iterations), where black dots represent the population distribution and red dots denote the optimal solution.

From the figure, it can be observed that at iteration 1, which represents the initial state, all population agents are randomly distributed across the entire search space. As the number of iterations increases, up to iteration 100, there is no significant aggregation of agents around the optimal solution, indicating that the algorithm maintains comprehensive global exploration in the early stages. By iteration 250, some agents have started moving toward the vicinity of the optimal solution. At iteration 500, all functions exhibit a noticeable concentration of agents around the optimal solution, demonstrating that in the middle and late stages of the iterations, the population shifts towards refined local exploitation to further optimize the solution.

Finally, in each iteration, the population diversity is quantified by computing the mean deviation of agents from the median in each dimension and then averaging these deviations across all dimensions [31]. This can be expressed by Equation (17):

(17) $$P\;D=\frac{1}{D}\sum_{j=1}^{D}\left(\frac{1}{N}\sum_{i=1}^{N}\left|x_{i,j}-M_{j}\right|\right)$$

where *PD* is the population diversity, *N* is the number of agents, *D* is the number of dimensions, x_i,j_ is the value of agent i in dimension *j*, and M*_j_* is the median of all agents in dimension j. These values are recorded and plotted as a curve, with the horizontal axis representing the number of iterations and the vertical axis representing the diversity value, reflecting the distribution changes of the population in the search space. The resulting curves are shown in column (f). Observing the curves in column (f), a common trend emerges across all functions: high diversity in the early stage, a rapid decline in the middle stage, and stabilization in the later stage. In the initial iterations, all function curves exhibit high population diversity, indicating that the use of the Fuch mapping results in a well-distributed initial population. High diversity implies significant differences between agents, facilitating global exploration. As the algorithm progresses, the population gradually converges toward the optimal solution, leading to a reduction in differences among agents. During this phase, the algorithm transitions from global exploration to local exploitation, accompanied by a decrease in population diversity. In the final convergence stage, the population becomes concentrated in a smaller region, with minimal differences among agents. These observed diversity trends confirm that the algorithm maintains good population diversity and convergence behavior.

## 5. Experiments and Analysis

To comprehensively and rigorously evaluate the performance of the algorithm proposed in this paper, we designed and conducted two sets of experiments in this section. A total of 16 comparative algorithms were selected, utilizing two widely used test suites. Detailed function information for these two test suites can be found in Table 6. Specific details, literature sources, and relevant parameters for all comparative algorithms discussed in Section 5 and Section 6 of this paper are available in Table 7.

The first set of experiments (detailed in Section 5.1) involved a comparative analysis of nine classic optimization algorithms on high-dimensional instances of the CEC2017 test suite. The second set of experiments (detailed in Section 5.2) evaluated seven state-of-the-art and highly relevant high-performance improved algorithms against the MSRIME algorithm on the latest CEC2022 test suite. Finally, to ensure the statistical significance of the evaluation results, we performed a statistical analysis of the average performance data from both experiment sets using the Wilcoxon rank-sum test and the Friedman test (detailed in Section 5.3).

To ensure fairness and reliability, all experiments are conducted under a unified environment: MATLAB 2019a as the software platform and an Intel(R) Core(TM) i7-8750H CPU @ 2.20 GHz 2.21 GHz as the hardware environment.

### 5.1. First Set of Experiments (CEC2017)

In this subsection, MSRIME is compared with nine highly cited optimization algorithms. These include traditional classical intelligent optimization algorithms such as Particle Swarm Optimization (PSO) [32], Gravitational Search Algorithm (GSA) [33], and Sparrow Search Algorithm (SSA) [34]. Additionally, high-performance optimization algorithms such as Grey Wolf Optimizer (GWO) [35] and Whale Optimization Algorithm (WOA) [36] are considered. Moreover, the comparison includes recently proposed optimization algorithms post-2021, namely the African Vulture Optimization Algorithm (AVOA) [37], Dung Beetle Optimization Algorithm (DBO) [38], Hunter-Prey Optimization Algorithm (HPO) [39], as well as the original RIME algorithm. The relevant parameters of these comparative algorithms are provided in Table 7.

**Figure 11:**
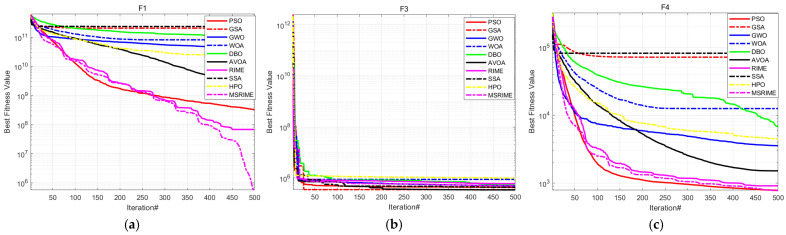

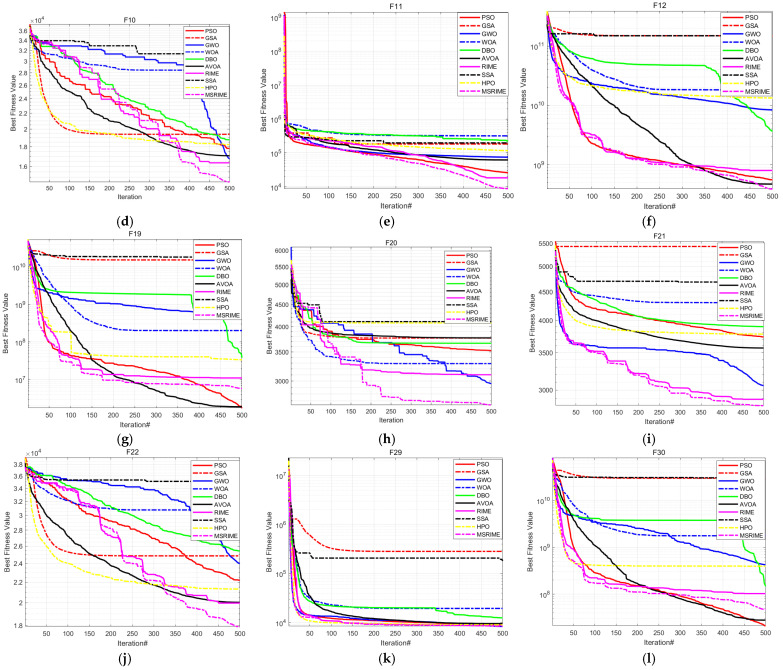
CEC2017 Test Convergence Curves: (**a**–**l**) represent the convergence curves of functions F1, F3, F4, F10, F11, F12, F19, F20, F21, F22, F29, and F30, respectively.

To comprehensively evaluate the optimization capability of MSRIME, experiments were conducted using the widely adopted CEC 2017 test suite at a dimensionality of D = 100. The population size was set to 50, with a maximum of 500 iterations. Function F2 was excluded, and the remaining 29 functions were tested. The optimization results were analyzed based on the average value (AVG) and standard deviation (STD) from 30 independent runs. The average value reflects the algorithm’s optimization ability, while the standard deviation indicates its stability. Table 8 presents the average values and standard deviations of the experimental results, and the convergence curves are illustrated in Figure 11, where “iteration#” denotes the number of iterations.

According to the experimental data in Table 8, even in the 100-dimensional case, the MSRIME algorithm, integrating multiple improvement strategies, achieves the best performance on most test functions. Although for functions F3, F11, F19, F29, and F30, the mean value of MSRIME does not reach the optimal level, it still demonstrates a significant advantage over the original algorithm, proving the effectiveness of the proposed improvements. Notably, when handling multimodal and hybrid functions, MSRIME exhibits the best average performance across all multimodal functions, highlighting its exceptional ability to escape local optima. Furthermore, for all hybrid functions except F19 and F20, MSRIME also achieves the best mean performance and, in most cases, the lowest standard deviation. This indicates that even when faced with complex problems, MSRIME can effectively approach the global optimum while maintaining high reliability.

To further analyze the algorithm’s iterative process, this study presents the convergence graphs for 12 selected functions, as shown in Figure 11. These 12 functions were chosen from the 29 test functions based on the following criteria: the first and last functions from the unimodal and multimodal categories, and the first two and last two functions from the hybrid and composition categories.

Observing the convergence curves, MSRIME consistently demonstrates a faster convergence speed on most functions. Notably, for functions F1, F10, and F22, MSRIME is able to escape local optima in subsequent iterations, leading to superior solutions. This indicates that the combination of the cosine annealing strategy and the adaptive search strategy effectively extends the algorithm’s exploration range. Furthermore, MSRIME exhibits the strongest convergence on the majority of function curves, which further confirms that the fusion of multiple strategies not only overcomes the limitations of the original algorithm but also significantly enhances optimization efficiency.

### 5.2. Second Set of Experiments (CEC2022)

This section presents the test analysis for the second set of comparative algorithms, utilizing the latest CEC 2022 test suite. This set specifically includes enhanced versions of the algorithms tested in Section 5.1, with a focus on recently proposed high-performance optimization methods.

The selected comparative algorithms include improved versions of the GWO algorithm: the SOGWO algorithm [40] and the IGWO algorithm [41], as well as an enhanced version of the WOA algorithm: the EWOA algorithm [42]. All these algorithms have been frequently cited in top-tier journals over the past five years. Additionally, we selected algorithms proposed within the last three years that share similarities with the MSRIME algorithm in terms of multi-strategy improvements. These include the Multi-Strategy Whale Optimization Algorithm (MSWOA) [43] and the Multi-Strategy Sparrow Search Algorithm (MISSA) [44]. Finally, to ensure a more targeted comparison with the proposed algorithm, the latest improved versions of the RIME algorithm, namely the IRIME algorithm [45] and the ACGRIME algorithm [46], were also chosen to more accurately assess the performance advantages of the proposed algorithm.

The test results are shown in Table 9. From the table, it can be observed that MSRIME exhibits outstanding performance on the vast majority of test functions, particularly on f1, f4, f5, and f11, where its mean value is significantly better than other algorithms. In terms of standard deviation, MSRIME achieves the smallest values on multiple functions, indicating high solution stability.

For the comparison algorithms: IGWO, as a highly cited improved version of GWO, performs well on certain functions (e.g., f3), but its overall performance is still slightly inferior to MSRIME. MSWOA, despite also employing multi-strategy improvement techniques, fails to surpass MSRIME in both mean values and standard deviations, with particularly noticeable gaps in f1, f4, and f7. MISSA, as a multi-strategy improved version of the Sparrow search algorithm, falls significantly short of MSRIME in overall performance. IRIME and ACGRIME, as improved versions of RIME, perform well on certain functions. In particular, ACGRIME achieves the best mean and standard deviation on f6 and f10. However, from the overall test results, MSRIME attains the optimal mean value in 2/3 of the functions, demonstrating significant advantages in optimizing complex functions. Even in functions where MSRIME does not achieve the best result, the gap between its performance and the best-performing algorithm is relatively small. Overall, MSRIME surpasses other comparison algorithms in terms of stability and optimization depth, making it a more reliable optimization method.

**Figure 12:**
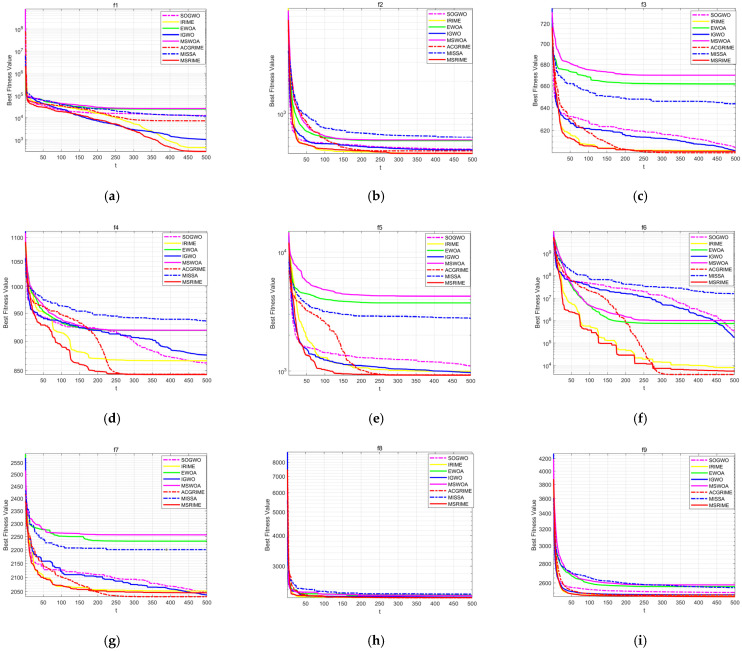

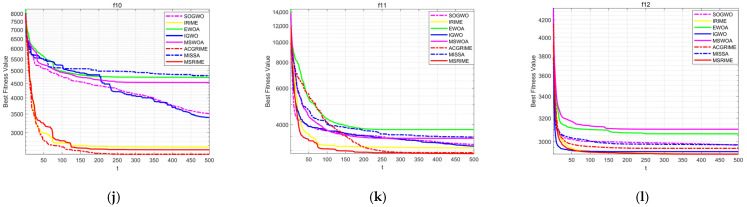
CEC2022 Test Convergence Curves: (**a**–**l**) present the convergence curves of functions f1-f12.

The convergence curves comparing the MSRIME algorithm with other high-performance improved algorithms are shown in Figure 12. From the convergence curves, it can be observed that, except for f10 in (j), MSRIME converges significantly faster than other algorithms in all other figures, achieving the optimal fitness value in the early iteration stage and maintaining stability thereafter. This phenomenon reflects that the introduction of the Fuch mapping and elite strategy control effectively improves the population quality in the early iteration stage, accelerating the convergence speed of the algorithm.

On the convergence curves of functions f4, f5, f6, f7, f10, and f11, the MSRIME algorithm exhibits multiple step-like drops in the mid-iteration stage. Similarly, on function f1, when the curve has already flattened in the late iteration stage, a significant jump-like drop is also observed. These phenomena are typical cases of escaping from the current local optimum, indicating that MSRIME possesses a superior ability to escape local optima. This further demonstrates that the combination of the cosine annealing strategy for adjusting the soft frost search step size and the adaptive search strategy’s dynamic adaptation of the search space effectively prevents the algorithm from getting trapped in local optima.

### 5.3. Statistical Significance Analysis

To statistically validate whether the optimization results of the improved MSRIME algorithm are superior to other comparative algorithms, we further employed the Wilcoxon signed-rank test and the Friedman test [47] for analysis in this section.

In the Wilcoxon test, three symbols (+/−/=) are used for evaluation: “+” indicates that MSRIME outperforms the other algorithm, “−” signifies that MSRIME’s performance is worse, and “=” denotes similar performance between MSRIME and the other algorithm. The Friedman test is utilized to assess the overall performance of the algorithms, where a lower ranking value indicates better performance.

Table 10 presents the Wilcoxon signed-rank test results and the average Friedman ranks for both sets of experiments conducted in Section 5.1 and Section 5.2. As shown in the table, the Wilcoxon signed-rank test results demonstrate that MSRIME exhibits a significant advantage in the vast majority of test functions, with the occurrence of “+” surpassing 2/3 of the comparisons. This indicates that, in terms of statistical significance, MSRIME’s optimization results are demonstrably superior to other algorithms, showcasing high reliability and stability.

**Figure 13:**
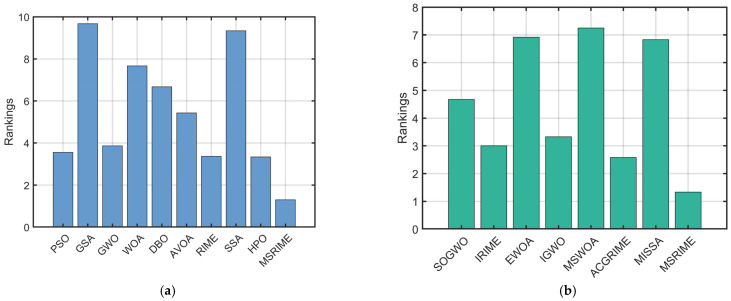
Friedman Test Results of Classical Algorithms. (**a**) CEC 2017. (**b**) CEC 2022.

Furthermore, the Friedman test results in Figure 13 and the rankings in Table 10 consistently show that MSRIME achieves the highest rank among all comparative algorithms, illustrating its overall strongest optimization capability. Therefore, based on this statistical analysis, it can be concluded that the proposed MSRIME algorithm outperforms other advanced optimization algorithms.

## 6. Path Planning for Delivery Robots Based on MSRIME

### 6.1. Robot Design and Application

**Figure 14:**
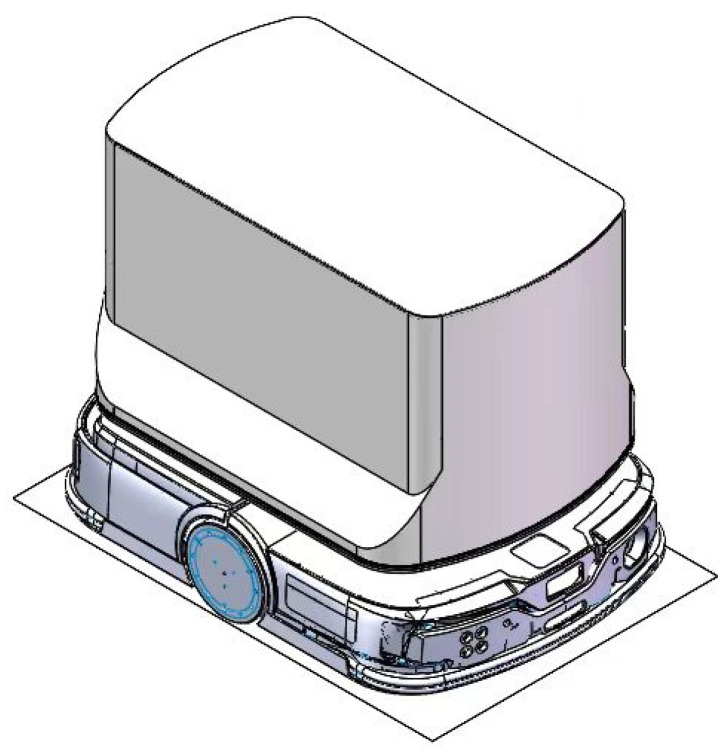
Construction of Delivery Robot.

As shown in Figure 14, the structural design of the delivery robot embodies a perfect combination of functionality and practicality. The robot primarily consists of two main parts: The upper section houses the storage compartment, which adopts a modular design that allows for flexible capacity adjustments based on the size and quantity of delivery items. The storage compartment is equipped with an intelligent lock system to ensure security during transportation. Additionally, it supports user authentication, enabling contactless delivery. The lower section contains the power base, which serves as the robot’s mobility core. The power base features high-performance drive motors and a rotating wheel assembly, providing precise path planning and long-lasting endurance, ensuring that the robot can navigate complex environments with agility.

As an automated mobile delivery robot, its primary task is to autonomously plan routes and efficiently transport items to designated locations. It can be deployed in various environments, including urban areas, campuses, and factories, catering to different architectural distributions. By offering convenient, efficient, and secure delivery services, the robot helps reduce labor costs and enhance operational efficiency.

The robot’s workflow consists of the following five stages:

(1) Initialization Stage: The robot departs from a designated starting location and completes system self-checks and position initialization.
(2) Loading Stage: The robot receives a delivery task, loads the items into the storage compartment, and confirms the item details and destination.
(3) Path Planning Stage: Based on the current environment and destination, the robot calculates an optimal route from its current position to the target location. The optimal route is determined by considering path length, travel time, energy consumption, and safety factors.
(4) Delivery Stage: The robot follows the planned route, detecting and avoiding obstacles during transit.
(5) Arrival Stage: Upon reaching the destination, the robot awaits item retrieval, confirms successful handover, and then returns to its starting point to prepare for the next delivery task.

Among all workflow stages, path planning is the core process that enables autonomous navigation. This crucial task is assigned to the MSRIME algorithm, ensuring efficient and adaptive route optimization for the delivery robot.

### 6.2. Adaptability Study of MSRIME in Path Planning

In path planning problems, if the map size is *h* rows and *m* columns, the problem dimension dim is *m* − 2. Each solution in the algorithm represents a vector of length dim. The fitness function is defined as shown in Equation (18), which includes the path evaluation function *Fl*, the turning point evaluation function *Fz*, and parameters *p* and *q* as weights for the two evaluation metrics. These weights can be set according to actual requirements. *Bn* represents the number of nodes on the path that are located on obstacles. The path evaluation function *Fl* is defined as the shortest path length, calculated as shown in Equation (19). The turning point evaluation function *Fz* is defined as the number of turns in the path. The calculation process first determines whether the *i*-th point on the path has a turn using Equations (21) and (22), and then computes *Fz* using Equation (20), where n represents the total number of points on the path, including the starting and ending points, and the coordinates of the *i*-th point are (*x_i_*,*y_i_*).

(18) $$F=\{\begin{array}{c} p\cdot Fl+q\cdot Fz\\ h\cdot m\cdot Bn \end{array}\begin{array}{c} ,Bn=0\\ ,Bn\neq 0 \end{array}$$

(19) $$Fl=\sum_{i=1}^{n-1}\sqrt{{\left(x_{i+1}-x_{i}\right)}^{2}+{\left(y_{i+1}-y_{i}\right)}^{2}}$$

(20) $$Fz=\sum_{i=2}^{n-1}fz$$

(21) $$fz=\{\begin{array}{c} 1,\\ 0, \end{array}\begin{array}{c} f_{t}(i)\neq f_{t}(i+1)\\ f_{t}(i)=f_{t}(i+1) \end{array}$$

(22) $$f_{t}(i)=\arctan(\frac{y_{i}-y_{i-1}}{x_{i}-x_{i-1}})$$

In optimization algorithms for delivery robot path planning, the variation in search step size significantly influences the coordinate changes of individuals during the iteration process, including front-to-back coordinate differences and adjacent dimension differences, thereby affecting the quality of the generated path. The front-to-back coordinate difference reflects the magnitude of an individual’s movement over consecutive iterations, and the search step size directly determines the variation amplitude of this difference. For a given node (*x_i_*,*y_i_*), its coordinate difference between iterations is denoted as Δ*d*, as shown in Equation (23), while the average front-to-back coordinate difference Δ*avg* is calculated using Equation (24), where old represents the coordinate before iteration, new represents the coordinate after iteration, *t* denotes the current iteration number, *T* is the total number of iterations, and *N* represents the population size. The adjacent dimension difference reflects the numerical difference between adjacent dimensions within the same iteration and is influenced by search mechanisms, initialization strategies, and step size variations. Its average value is computed using Equation (25), where *R*_*i*,*j*_ (*t*) represents the coordinate value of the *i*-th individual in the *j*-th dimension during the *t*-th iteration.

As described in Section 2.2 and Section 3.4, the soft frost search step size in the RIME algorithm follows a stepwise variation with multiple step jump points. When a step jump occurs, the magnitude of an individual’s iterative movement significantly increases, directly leading to a larger front-to-back coordinate difference Δ*d*. Meanwhile, at the jump points, the variation amplitude of certain dimensions also increases considerably, causing a rise in the average adjacent dimension difference Δ*r*, thereby increasing the number of turning points in the path and making the path more tortuous.

(23) $$\Delta d_{i}(t)=\left|x_{i}^{new}(t)-x_{i}^{old}(t)\right|+\left|y_{i}^{new}(t)-y_{i}^{old}(t)\right|$$

(24) $$\Delta avg=\frac{1}{2N\cdot T}\sum_{t=1}^{T}\sum_{i=1}^{N}\Delta d_{i}(t)$$

(25) $$\Delta r=\frac{\sum_{t=1}^{T}\sum_{i=1}^{N}\sum_{j=2}^{\dim}\left|R_{i,j}(t)-R_{i,j-1}(t)\right|}{T\cdot (\dim-1)\cdot N}$$

By introducing the Fuch chaotic mapping, controlled elite strategy, and adaptive search strategy, the MSRIME algorithm significantly enhances early-stage search capability and overall optimization performance, effectively improving the global optimality of path planning and better meeting the practical requirements of delivery robot applications. Furthermore, to address the issue in the RIME algorithm where stepwise changes in step size may degrade path quality, MSRIME adopts a cosine annealing strategy, ensuring a smooth and continuous variation of step size during iterations. This prevents the increase in front-to-back coordinate differences and adjacent dimension differences caused by abrupt step jumps, thereby enhancing the stability and quality of path planning.

**Figure 15:**
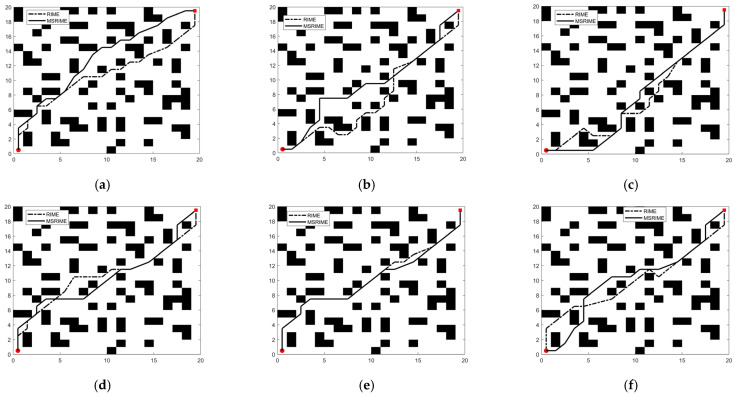
Path Planning Results: (**a**–**f**) sequentially display the path planning experimental results from the first to the sixth trial.

To validate the effectiveness of these improvements and further analyze the applicability of MSRIME compared to RIME in the field of path planning, this study conducts six path planning experiments using RIME and MSRIME algorithms on a randomly generated grid map (as described in Section 6.3). In the experiments, the average front-to-back coordinate difference Δ*avg* and the average adjacent dimension difference Δ*r* are computed using Equations (24) and (25), respectively, while path length *L* and the number of turning points *z* are obtained using Equations (19) and (20). The experimental results are shown in Figure 15, with relevant statistical data summarized in Table 11.

From the experimental results, in the six tests conducted, the path length *L* obtained by the MSRIME algorithm was superior to or equal to that of the RIME algorithm in five tests, while the number of turning points *z* was superior to or equal to that of RIME in four tests. The average front-back coordinate difference Δ*avg* was superior to or equal to RIME in four tests, and the average adjacent-dimension difference Δ*r* was superior to or equal to RIME in five tests. This indicates that MSRIME can generate smoother and higher-quality paths in most cases.

Notably, in experiments b, c, and e, smaller values of Δ*avg* and Δ*r* were indeed more conducive to producing smooth and high-quality paths. These findings suggest that though the average front-back coordinate difference and the average adjacent-dimension difference do not directly determine the path length *L* and the number of turning points *z*, they do exert a certain influence on these metrics. Smaller values of Δ*avg* and Δ*r* contribute to more uniform changes in path nodes, reducing abrupt turning points and enhancing both path smoothness and global optimality.

Overall, the MSRIME algorithm not only inherits the high computational efficiency of the RIME algorithm but also introduces a single control factor, further simplifying the implementation of multiple strategies and enhancing the algorithm’s operability and stability. More importantly, MSRIME demonstrates outstanding overall optimization capability, exhibiting significant advantages over the RIME algorithm in key metrics such as global optimality in path planning, path smoothness, and path length. These results preliminarily validate MSRIME’s superior adaptability in delivery robot path planning applications, indicating that this method can generate high-quality paths more effectively.

Furthermore, the experiments in Section 6.4 will further validate these conclusions and explore the application potential of the MSRIME algorithm in delivery robot path planning, aiming to comprehensively evaluate its actual performance in different environments.

### 6.3. Environment Setup

In this experimental setup, grid-based modeling is employed for environment representation. This method divides the entire environment into a series of uniform grid cells, facilitating path planning and analysis. Each cell in the grid map represents a specific location in the environment and is labeled based on its traversability: Traversable grid cells are marked in white, non-traversable grid cells (occupied by buildings) are marked in black. Grid cells are indexed systematically according to their coordinates, following a left-to-right and bottom-to-top ordering. This systematic indexing provides a clear structure for path planning, ensuring efficient navigation.

To enhance the realism of the simulation, the delivery robot’s map is designed based on building density and Building Coverage Ratio (BCR). The BCR is defined as the ratio of building area to land area and is commonly used to assess urban building scale and planning scope [48].

Taking the United States as an example, Soliman et al. [49] conducted a statistical analysis of the geographical distribution of BCR across various regions in the U.S. and provided detailed BCR information for the city of Chicago. The results show that the BCR in Chicago ranges from 0% to 83%, with over 75% of the areas having a BCR between 7% and 26%. Therefore, the BCR for the first map in this experiment is set within the range of 7% to 26% to reflect the most common building density. Additionally, Le et al. [50] studied the building density and BCR in highly urbanized areas, finding that the BCR in such regions varies widely, ranging from 40% to 80%. However, Lau et al. [51] pointed out that every 10% increase in building coverage can lead to a temperature rise of 0.28 °C, and a BCR exceeding 50% can impose significant burdens on the urban environment, traffic, and energy consumption. As a result, with the optimization of urban planning, new cities tend to reduce building coverage to mitigate the heat island effect and improve the urban environment. Particularly in path planning experiments, if the BCR is too high, the number of feasible paths will significantly decrease, which is unfavorable for testing algorithm performance, especially in small-scale maps. Based on this, the BCR for the second map in this experiment is set to be higher than 26% but below 50%, with the BCR for small-scale maps slightly lower than that for large-scale maps.

To comprehensively evaluate the path planning capability of the MSRIME algorithm in various delivery environments, four simulated environments with different building densities and map scales were designed based on the above analysis. The detailed specifications are presented in Table 12.

The experiment was designed with two typical regions: general areas and urbanized areas. In general areas, obstacles account for 26% of the total area and are randomly distributed. In contrast, in urbanized areas, the number of obstacles significantly increases to 40%, with a more complex layout, simulating intricate urban road conditions. The experiment maps are categorized into two scales: small-scale maps and large-scale maps. The small-scale map has a size of 20 × 20, with the starting point at (0,0) and the destination at (20,20). The large-scale map has a size of 40 × 40, with the starting point at (0,0) and the destination at (40,40). To reflect the difference in obstacle density across map scales, the obstacle proportion in small-scale maps is 5% lower than that in large-scale maps.

To comprehensively evaluate the performance of path planning, metrics such as path length *L* (Equation (19)), execution time *time*, and the number of path turning points *z* (Equation (20)) are adopted to assess energy consumption, decision-making efficiency, path feasibility, and algorithm superiority, respectively. A shorter path length and fewer turning points indicate higher path quality.

In this experiment, the comparison algorithms include the classical algorithms from Section 4, namely GWO, RIME, and DBO; the high-performance improved algorithms SOGWO and IRIME; as well as the optimization algorithms specifically designed for robot path planning, FSA and ISSA, as mentioned in references [10,13]. The number of experiments is set to 30, and the remaining parameters remain consistent with Section 5.

To quantify the performance improvement of MSRIME compared to the original RIME algorithm, this study calculates the performance variation rate α, which serves as a measure of the overall optimization efficiency of the improved algorithm. The specific calculation method for *α* is given in Equation (26), where *Q* represents various performance indicators such as *L*, *time*, and *z*. *Q*_MSIME_ denotes the experimental results of the MSRIME algorithm under the corresponding indicator, while *Q*_RIME_ represents the results of the RIME algorithm. A larger *α* value indicates a more significant performance improvement of the algorithm.

(26) $$\alpha_{Q}=\frac{\left|Q_{MSRIME}-Q_{RIME}\right|}{Q_{RIME}}\times 100\%$$

### 6.4. Results Presentation

**Figure 16:**
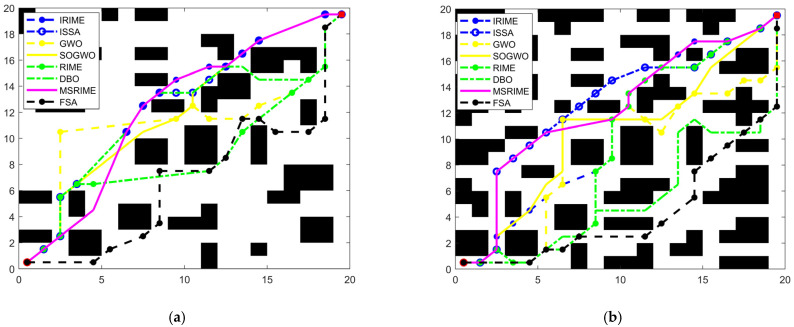
Experimental results of small-scale map path planning. (**a**) Small ordinary map. (**b**) Small urbanization Map.

The experimental results for the small-scale maps are shown in Figure 16, with the corresponding summary of various performance indicators provided in Table 13. From the analysis of the images and tables, it is evident that different algorithms exhibit varying path planning strategies, regardless of whether they are applied to ordinary maps or urban maps.

In terms of final results, the MSRIME algorithm consistently achieves the optimal path length across all tested environments. Although the MSRIME algorithm does not achieve the shortest average path length in urban maps, it is only 0.4728 units longer than the ISSA algorithm, which ranks first, indicating a minor gap in path length performance. Notably, in the small-scale urban map, MSRIME achieves the best performance across all indicators except execution time. However, the execution time difference between MSRIME and the fastest algorithm is only 0.0622 s, demonstrating its competitive efficiency.

From the overall experimental results, MSRIME exhibits significant advantages in multiple aspects of path planning tasks, including path quality and planning efficiency.

**Figure 17:**
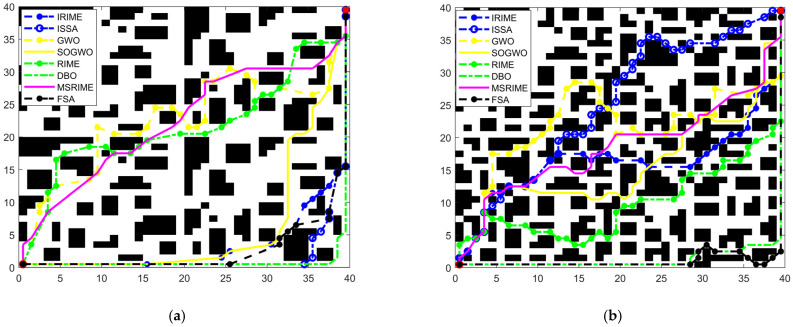
Experimental results of large-scale map path planning. (**a**) Large ordinary map. (**b**) Large urbanization map.

The experimental results for the large-scale maps are shown in Figure 17, with the corresponding summary of various performance indicators provided in Table 14. Due to the lower building coverage ratio (BCR) in the ordinary map, the obstacle distribution is relatively sparse, and the robot rarely encounters complex impassable segments. As a result, the path planning results in fewer turning points. In contrast, in the urban map, where the BCR is higher, there are fewer passable routes and denser obstacles, requiring the robot to navigate around many obstacles, leading to a significant increase in turning points during path planning.

From the analysis of images and tables, it is clear that the MSRIME algorithm achieves the best results in terms of average path length in both the ordinary and urban maps. It also performs best in terms of optimal path length in the ordinary map. While the MSRIME algorithm’s optimal path length in the urban map is slightly worse than that of the ISSA algorithm, it significantly outperforms the ISSA algorithm in terms of execution time and path smoothness. This indicates that the MSRIME algorithm can generate smoother paths in less time, reducing the number of turns the robot has to make, which in turn improves motion efficiency and reduces energy consumption.

In conclusion, even when facing complex and variable road conditions in the large-scale map, the MSRIME algorithm is able to efficiently find optimal paths. Its outstanding global search ability, fast convergence performance, and good path smoothness give it a significant advantage in path planning tasks in complex environments.

**Figure 18:**
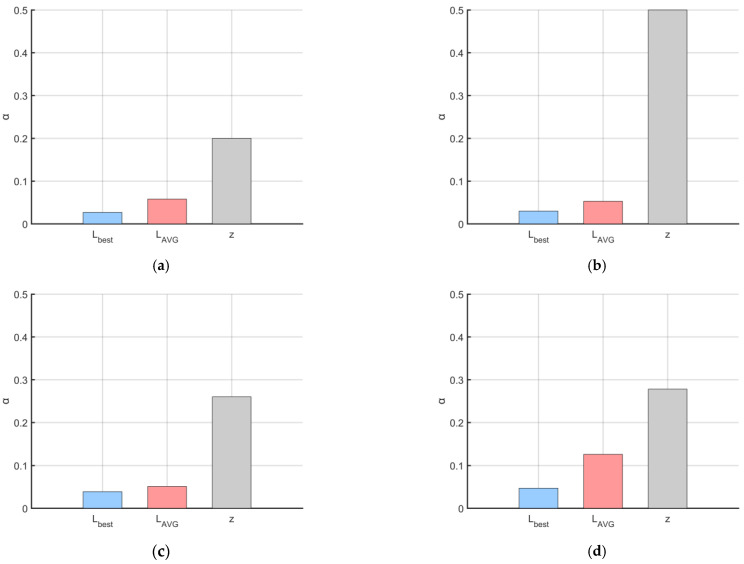
Performance change rate results. (**a**) Small ordinary map. (**b**) Small urbanization map. (**c**) Large ordinary map. (**d**) Large urbanization map.

Since the MSRIME algorithm introduces additional computational complexity compared to the RIME algorithm to enhance its optimization capability, its execution time does not show a significant advantage over RIME. Across the four sets of experiments, the execution time of MSRIME remains within ±0.1 s of that of RIME, indicating a minimal difference in computational efficiency. Thus, when calculating the performance change rate (*α*), only optimal path length, average path length, and number of path turning points were selected as evaluation metrics. The final results are presented in Figure 18.

From Figure 18, it can be observed that all three indicators show a positive change across all four maps, confirming that the overall planning capability of MSRIME has significantly improved compared to RIME. Specifically, Average path length improved by 5.8%, 5.3%, 5.1%, and 12.6%, respectively. Number of path turning points reduced by 20%, 50%, 26%, and 27.8%, respectively. This indicates that under the guidance of the MSRIME algorithm, the robot can quickly adapt to different environments, plan smoother paths with fewer turning points, and minimize path length, leading to significant reductions in energy consumption.

Notably, the MSRIME algorithm excels in reducing path turning points. In the small-scale urban map, the number of turning points decreased by 50%, demonstrating effective turning point optimization, which directly enhances the robot’s overall movement efficiency. This result further reinforces the practical applicability of the MSRIME algorithm in path planning tasks.

### 6.5. Additional Tests for Multi-Scenario Application

Building upon the systematic validation of the delivery robot’s path planning capabilities across various BCR scenarios in Section 6.4, this section designs four distinct and functionally differentiated test scenarios for additional experiments, aiming to further investigate the algorithm’s adaptability in special environments.

Scenario 1 simulates a 20 × 20 warehouse environment, constructing a shelf-shuttling situation with five standard shelves and randomly distributed small obstacles, primarily examining the robot’s ability to navigate around shelves. Scenario 2 replicates a loop-shaped corridor structure commonly found in buildings such as schools or hotels, enhancing navigation difficulty through random obstacle placement. Scenario 3 constructs a large, winding passage, simulating complex road networks within logistics parks, where the starting coordinates are (0, 10) and the ending coordinates are (40, 30), Scenario 4 deploys large elliptical obstacles on a 40 × 40 map, effectively simulating impassable barriers in natural environments like ponds, rock formations, or dense thickets.

**Figure 19:**
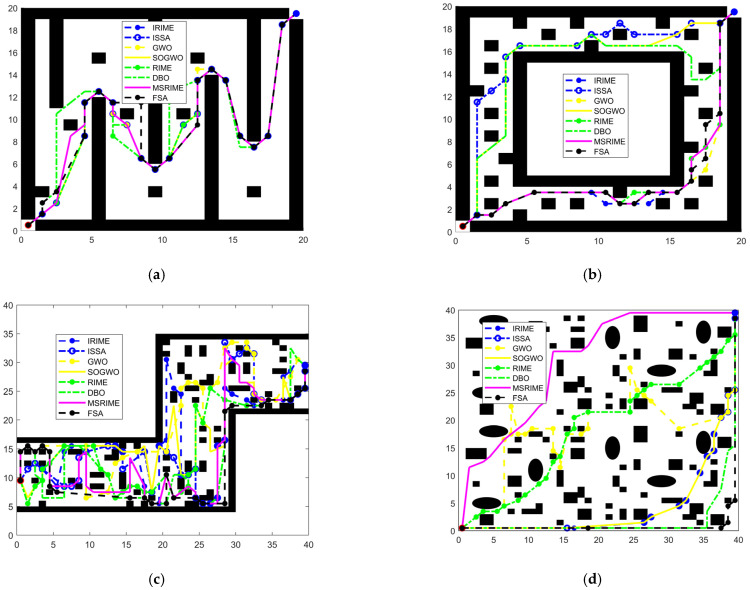
Additional test results. (**a**) Scenario 1. (**b**) Scenario 2. (**c**) Scenario 3. (**d**) Scenario 4.

Through this rigorously designed set of comparative experiments, we evaluate the robust path planning performance of the MSRIME algorithm in multimodal special scenarios. The experimental results are presented in Figure 19 and Table 15.

Analysis of the experimental data from Figure 19 and Table 15 reveals that across the four test scenarios, Scenarios 1 and 2 exhibited relatively minor performance differences among the algorithms due to the limited traversable paths. Nevertheless, the MSRIME algorithm secured a close second place in Scenario 2 for average path length, while maintaining a leading edge in crucial metrics like optimal path length and number of turns.

Particularly in the tests for Scenarios 3 and 4, although the MSRIME algorithm was slightly inferior to the DBO and FSA algorithms in terms of the number of turns in Scenario 4, its performance in the two core metrics—average path length and optimal path length—significantly surpassed both algorithms, fully demonstrating its performance advantage.

Regarding computational efficiency, the MSRIME algorithm retained the high-efficiency characteristics of the RIME algorithm, introducing only an additional time overhead of less than 0.2 s while achieving a comprehensive improvement across all performance indicators.

Cumulatively, across all four scenarios, the MSRIME algorithm achieved optimal performance in 75% of the test scenarios for the three key metrics: optimal path length, average path length, and number of turns. This result powerfully validates the algorithm’s outstanding adaptability and robustness in multi-scenario path planning tasks.

## 7. Conclusions

To address the prevalent issues of local optima, low computational efficiency, and poor adaptability in complex dynamic environments often encountered by traditional path planning algorithms, this paper proposes a Multi-Strategy Controlled Rime Optimization Algorithm (MSRIME). Unlike existing mainstream multi-strategy improvement algorithms, MSRIME innovatively presents a multi-strategy collaborative framework that relies on a control factor to coordinate and complement various strategies. This framework incorporates multiple effective experiments, such as chaotic characteristic analysis and population variation analysis, in the selection of individual strategies, thereby achieving a performance enhancement where the whole is greater than the sum of its parts.

In simulation experiments, MSRIME’s significant advantages were demonstrated through optimization comparison experiments and statistical analysis. Subsequently, this paper delves into the impact of coordinate differences between successive nodes and differences in adjacent dimensions within multi-dimensional space on path quality, verifying that the MSRIME algorithm can effectively mitigate these effects, thereby improving path quality. Finally, in the path planning experiments, to better align with real-world obstacle distributions, this paper analyzed the Building Coverage Ratio (BCR) design ranges of mainstream architectural environments and, for the first time, applied them to the map settings for delivery robot path planning simulation experiments. The results confirmed MSRIME’s advantages in key metrics such as path length, runtime, and path smoothness, proving its effectiveness in planning shorter, smoother, and more energy-efficient routes. This further substantiates MSRIME’s reliability in supporting path planning for delivery robots in practical applications.

The multi-strategy control framework proposed in this paper, along with the analysis of the impact of coordinate differences between successive nodes and differences in adjacent dimensions within multi-dimensional space on path quality, not only offers an alternative algorithm for smart logistics but also provides new insights for future bionic algorithm improvements and advancements in the field of path planning. However, this study still has certain limitations: first, global path planning heavily relies on precise environmental maps, which may lead to performance degradation in real-world applications due to map update delays or sensor errors; second, this paper exclusively focuses on two-dimensional path planning, without considering multi-level buildings or complex three-dimensional terrains, thus limiting the validation of the algorithm’s scalability; third, experiments were conducted solely in a simulated environment, lacking verification on physical hardware platforms, which means real-world deployment performance remains untested. Future research will address these limitations by exploring MSRIME’s applications in dynamic environments, 3D spaces, and actual hardware platforms, thereby further enhancing its practicality and robustness.

## Figures and Tables

**Table 1:** Statistical Analysis of Chaotic Mappings.

Name	Dimension	Frequency of Occurrence	Name	Dimension	Frequency of Occurrence
Logistic	one-dimensional	204	Cubic	one-dimensional	32
Tent	one-dimensional	226	Kent	one-dimensional	14
Chebyshev	one-dimensional	25	Piecewise	one-dimensional	20
Henon	multi-dimensional	32	Lozi	multi-dimensional	5
Circle	one-dimensional	41	Fuch	one-dimensional	7

**Table 2:** Initial Value Sensitivity Analysis.

Initial Value	Variation Range	Logistic/%	Tent/%	Henon/%	Fuch/%
0.13	10^−6^	26.25	28.43	18.69	48.33
0.15	10^−6^	26.00	34.37	18.97	37.51
0.18	10^−6^	28.13	21.88	21.47	43.50
0.20	10^−6^	26.25	28.13	17.72	34.38
0.23	10^−6^	37.50	33.56	13.97	28.13
0.27	10^−6^	31.25	34.37	16.53	37.50

**Table 3:** Lyapunov Exponent Analysis.

Chaotic Mapping	Logistic	Tent	Fuch	Henon
Lyapunov value	0.6941	0.7646	2.8661	0.4312

**Table 4:** Comparison of Chaotic Mappings.

F	FRIME	RIME	TRIME
F1	[**1.61 × 10^6^,4.37 × 10^7^**]	[3.91 × 10^6^,9.69 × 10^7^]	[9.16 **×** 10^6^,6.68 × 10^7^]
F2	[**1.91 × 10^87^**,1.98 × 10^113^]	[3.42 × 10^88^,**1.00 × 10^103^**]	[5.41 × 10^88^,5.10 × 10^114^]
F3	[**7.85 × 10^4^**,**1.90 × 10^5^**]	[8.76 × 10^4^,2.41 × 10^5^]	[7.97 × 10^4^,2.42 × 10^5^]
F4	[4.48 × 10^2^,**5.61 × 10^2^**]	[4.77 × 10^2^,6.28 × 10^2^]	[**4.37 × 10^2^**,5.80 × 10^2^]
F5	[**5.68 × 10^2^**,6.92 × 10^2^]	[5.81 × 10^2^,**6.60 × 10^2^**]	[5.93 × 10^2^,7.08 × 10^2^]
F6	[6.10 × 10^2^,**6.28 × 10^2^**]	[6.16 × 10^2^,6.35 × 10^2^]	[**6.09 × 10^2^**,6.30 × 10^2^]
F7	[**8.47 × 10^2^,9.72 × 10^2^**]	[8.53 × 10^2^,9.93 × 10^2^]	[8.76 × 10^2^,1.01 × 10^3^]
F8	[8.83 × 10^2^,9.91 × 10^2^]	[8.66 × 10^2^,**9.62 × 10^2^**]	[**8.65 × 10^2^**,9.81 × 10^2^]
F9	[**1.65 × 10^3^**,1.46 × 10^4^]	[2.28 × 10^3^,**9.45 × 10^3^**]	[1.97 × 10^3^,1.22 × 10^4^]
F10	[**3.78 × 10^3^**,6.43 × 10^3^]	[4.51 × 10^3^,6.77 × 10^3^]	[3.83 × 10^3^,**6.35 × 10^3^**]

**Table 5:** Multi-parameter and multi-strategy comparison experiment.

	MSRIME	RIMEa1	RIMEa2	RIMEa3	RIME1	RIME2	RIME3
AVG	5.14 × 10^3^	6.72 × 10^3^	7.49 × 10^3^	8.36 × 10^3^	7.47 × 10^3^	7.80 × 10^3^	5.77 × 10^3^
STD	1.73 × 10^3^	2.23 × 10^3^	2.57 × 10^3^	2.29 × 10^3^	2.25 × 10^3^	1.83 × 10^3^	1.96 × 10^3^

**Table 6:** Test Functions.

		Function Information		F_min_
CEC 2017	Unimodal Function	F1	Shifted and Rotated Bent Cigar Function	100
		F2	Shifted and Rotated Sum of Different Power Function	200
		F3	Shifted and Rotated Zakharov Function	300
	Simple Multimodal Functions	F4	Shifted and Rotated Rosenbrock’s Function	400
		F5	Shifted and Rotated Rastrigin’s Function	500
		F6	Shifted and Rotated Expanded Scaffer’s F6 Function	600
		F7	Shifted and Rotated Lunacek Bi_Rastrigin Function	700
		F8	Shifted and Rotated Non-Continuous Rastrigin’s Function	800
		F9	Shifted and Rotated Levy Function	900
		F10	Shifted and Rotated Schwefel’s Function	1000
	Hybrid Functions	F11	Hybrid Function 1 (*n* = 3)	1100
		F12	Hybrid Function 2 (*n* = 3)	1200
		F13	Hybrid Function 3 (*n* = 3)	1300
		F14	Hybrid Function 4 (*n* = 4)	1400
		F15	Hybrid Function 5 (*n* = 4)	1500
		F16	Hybrid Function 6 (*n* = 4)	1600
		F17	Hybrid Function 6 (*n* = 5)	1700
		F18	Hybrid Function 6 (*n* = 5)	1800
		F19	Hybrid Function 6 (*n* = 5)	1900
		F20	Hybrid Function 6 (*n* = 6)	2000
	Composition Functions	F21	Composition Function 1 (*n* = 3)	2100
		F22	Composition Function 2 (*n* =3)	2200
		F23	Composition Function 3 (*n* =4)	2300
		F24	Composition Function 4 (*n* = 4)	2400
		F25	Composition Function 5 (*n* = 5)	2500
		F26	Composition Function 6 (*n* = 5)	2600
		F27	Composition Function 7 (*n* = 6)	2700
		F28	Composition Function 8 (*n* = 6)	2800
		F29	Composition Function 9 (*n* = 3)	2900
		F30	Composition Function 10 (*n* =3)	3000
CEC 2022	Unimodal Function	f1	Shifted and full Rotated Zakharov Function	300
	Basic Functions	f2	Shifted and full Rotated Rosenbrock’s Function	400
		f3	Shifted and full Rotated Expanded Schaffer’s *f*6 Function	600
		f4	Shifted and full Rotated Non-Continuous Rastrigin’s Function	800
		f5	Shifted and full Rotated Levy Function	900
	Hybrid Functions	f6	Hybrid Function 1 (*n* = 3)	1800
		f7	Hybrid Function 2 (*n* = 6)	2000
		f8	Hybrid Function 3 (*n* = 5)	2200
	Composition Functions	f9	Composition Function 1 (*n* = 5)	2300
		f10	Composition Function 2 (*n* = 4)	2400
		f11	Composition Function 3 (*n* = 5)	2600
		f12	Composition Function 4 (*n* = 6)	2700
	Search Range: [−100, 100]			

**Table 7:** Algorithm parameter settings.

Algorithm	Reference	Full Algorithm Name	Parameter Information
FSA	[10]	Flamingo Search Algorithm	*b* = 0.1
ISSA	[13]	Improved sparrow search algorithm	*p* = 0.2
RIME	[15]	Rime algorithm	*w* = 5
PSO	[32]	Particle swarm optimization	*c*_1_ = *c*_2_ = 2, *w* = 0.7
GSA	[33]	Gravitational search algorithm	*α* = 20
SSA	[34]	Sparrow search algorithm	*p* = 0.2
GWO	[35]	Grey wolf optimizer	*α* = [2,0]
WOA	[36]	Whale optimization algorithm	*b* = 1
AVOA	[37]	African vultures optimization algorithm	*p* = 0.5
DBO	[38]	Dung beetle optimizer	*p* = 0.2
HPO	[39]	Hunter–prey optimization:	——
SOGWO	[40]	Selective opposition based grey wolf optimization	——
IGWO	[41]	Improved grey wolf optimizer	——
EWOA	[42]	Enhanced whale optimization algorithm	*b* = 1
MSWOA	[43]	Multi-strategy whale optimization algorithm	*b* = 1
MISSA	[44]	Multi-strategy improved sparrow search algorithm	*p* = 0.3
IRIME	[45]	Improved RIME optimization algorithm	*w* = 5
ACGRIME	[46]	Adaptive chaotic Gaussian RIME optimizer	*a* = 4

**Table 8:** CEC2017 Test Results.

	**F1**		**F3**		**F4**		**F5**		**F6**	
	**AVG**	**STD**	**AVG**	**STD**	**AVG**	**STD**	**AVG**	**STD**	**AVG**	**STD**
PSO	3.27 × 10^8^	6.36 × 10^7^	4.67 × 10^5^	8.52 × 10^4^	7.83 × 10^2^	1.31 × 10^2^	1.33 × 10^3^	7.58 × 10	6.70 × 10^2^	4.67
GSA	2.06 × 10^11^	1.04 × 10^10^	3.59 × 10^5^	2.63 × 10^4^	7.14 × 10^4^	8.25 × 10^3^	1.49 × 10^3^	1.40 × 10^2^	6.73 × 10^2^	3.43
GWO	4.32 × 10^10^	1.10 × 10^10^	4.75 × 10^5^	6.76 × 10^4^	3.56 × 10^3^	1.30 × 10^3^	1.17 × 10^3^	8.30 × 10	6.39 × 10^2^	3.53
WOA	8.18 × 10^10^	1.22 × 10^10^	1.07 × 10^6^	1.93 × 10^5^	1.25 × 10^4^	2.37 × 10^3^	1.79 × 10^3^	1.20 × 10^2^	6.97 × 10^2^	**2.30**
DBO	9.60 × 10^10^	7.72 × 10^10^	7.58 × 10^5^	3.07 × 10^5^	6.69 × 10^3^	4.59 × 10^3^	1.63 × 10^3^	1.80 × 10^2^	6.75 × 10^2^	1.21 × 10
AVOA	3.11 × 10^9^	1.60 × 10^9^	**3.30 × 10^5^**	**1.99 × 10^4^**	1.52 × 10^3^	2.06 × 10^2^	1.36 × 10^3^	8.41 × 10	6.68 × 10^2^	2.42
RIME	6.82 × 10^7^	2.50 × 10^7^	6.63 × 10^5^	5.93 × 10^4^	9.12 × 10^2^	1.10 × 10^2^	1.06 × 10^3^	8.88 × 10	6.50 × 10^2^	6.77
SSA	2.34 × 10^11^	4.39 × 10^10^	4.79 × 10^5^	4.93 × 10^4^	8.16 × 10^4^	1.10 × 10^4^	2.16 × 10^3^	8.51 × 10	7.11 × 10^2^	8.62
HPO	2.19 × 10^10^	1.93 × 10^10^	7.56 × 10^5^	1.26 × 10^5^	4.47 × 10^3^	2.85 × 10^3^	1.64 × 10^3^	1.56 × 10^2^	6.79 × 10^2^	9.71
MSRIME	**5.51 × 10^5^**	**2.84 × 10^5^**	4.34 × 10^5^	6.80 × 10^4^	**7.78 × 10^2^**	**5.86 × 10**	**9.84 × 10^2^**	**6.95 × 10**	**6.33 × 10^2^**	5.24
	**F7**		**F8**		**F9**		**F10**		**F11**	
	**AVG**	**STD**	**AVG**	**STD**	**AVG**	**STD**	**AVG**	**STD**	**AVG**	**STD**
PSO	2.34 × 10^3^	2.64 × 10^2^	1.73 × 10^3^	8.39 × 10	6.29 × 10^4^	1.00 × 10^4^	1.83 × 10^4^	9.80 × 10^2^	**8.58 × 10^3^**	3.03 × 10^3^
GSA	3.18 × 10^3^	2.07 × 10^2^	2.01 × 10^3^	**3.40 × 10**	3.22 × 10^4^	5.35 × 10^3^	2.07 × 10^4^	1.33 × 10^3^	1.84 × 10^5^	2.06 × 10^4^
GWO	1.96 × 10^3^	8.18 × 10	1.52 × 10^3^	6.98 × 10	3.81 × 10^4^	1.59 × 10^4^	2.01 × 10^4^	7.22 × 10^3^	7.51 × 10^4^	1.95 × 10^4^
WOA	3.74 × 10^3^	2.14 × 10^2^	2.27 × 10^3^	7.30 × 10	9.04 × 10^4^	2.45 × 10^4^	2.86 × 10^4^	1.34 × 10^3^	3.19 × 10^5^	1.64 × 10^5^
DBO	2.76 × 10^3^	2.50 × 10^2^	2.27 × 10^3^	2.51 × 10^2^	6.63 × 10^4^	7.43 × 10^3^	2.20 × 10^4^	5.23 × 10^3^	2.22 × 10^5^	5.92 × 10^4^
AVOA	3.12 × 10^3^	1.54 × 10^2^	1.77 × 10^3^	9.58 × 10	2.72 × 10^4^	**2.85 × 10^3^**	1.62 × 10^4^	1.85 × 10^3^	6.21 × 10^4^	1.99 × 10^4^
RIME	1.86 × 10^3^	1.22 × 10^2^	1.41 × 10^3^	9.59 × 10	3.82 × 10^4^	1.71 × 10^4^	1.61 × 10^4^	1.48 × 10^3^	1.84 × 10^4^	4.20 × 10^3^
SSA	4.61 × 10^3^	4.88 × 10^2^	2.58 × 10^3^	8.98 × 10	9.63 × 10^4^	1.42 × 10^4^	3.18 × 10^4^	1.40 × 10^3^	1.98 × 10^5^	3.06 × 10^4^
HPO	5.55 × 10^3^	6.67 × 10^2^	1.95 × 10^3^	2.03 × 10^2^	4.50 × 10^4^	9.16 × 10^3^	1.91 × 10^4^	1.13 × 10^3^	1.17 × 10^5^	5.65 × 10^4^
MSRIME	**1.63 × 10^3^**	**7.96 × 10**	**1.31 × 10^3^**	8.06 × 10	**2.05 × 10^4^**	4.25 × 10^3^	**1.59 × 10^4^**	**9.75 × 10^2^**	1.66 × 10^4^	**2.63 × 10^3^**
	**F12**		**F13**		**F14**		**F15**		**F16**	
	**AVG**	**STD**	**AVG**	**STD**	**AVG**	**STD**	**AVG**	**STD**	**AVG**	**STD**
PSO	5.54 × 10^8^	3.08 × 10^8^	1.40 × 10^6^	4.38 × 10^5^	2.97 × 10^6^	1.53 × 10^6^	1.41 × 10^5^	8.39 × 10^4^	6.36 × 10^3^	3.29 × 10^2^
GSA	1.49 × 10^11^	1.35 × 10^10^	3.40 × 10^10^	1.27 × 10^9^	3.57 × 10^7^	2.34 × 10^7^	1.35 × 10^10^	1.83 × 10^9^	1.87 × 10^4^	9.55 × 10^2^
GWO	8.39 × 10^9^	3.58 × 10^9^	1.19 × 10^9^	9.80 × 10^8^	1.16 × 10^7^	3.96 × 10^6^	2.05 × 10^8^	3.05 × 10^8^	6.87 × 10^3^	**1.67 × 10^2^**
WOA	1.83 × 10^10^	3.40 × 10^9^	1.05 × 10^9^	5.26 × 10^8^	1.39 × 10^7^	6.19 × 10^6^	1.42 × 10^8^	1.16 × 10^8^	1.65 × 10^4^	1.66 × 10^3^
DBO	3.64 × 10^9^	1.36 × 10^9^	9.63 × 10^7^	6.92 × 10^7^	1.26 × 10^7^	1.29 × 10^7^	1.01 × 10^7^	1.60 × 10^7^	8.81 × 10^3^	1.81 × 10^3^
AVOA	4.69 × 10^8^	2.85 × 10^8^	7.01 × 10^4^	2.40 × 10^4^	7.52 × 10^6^	4.06 × 10^6^	5.72 × 10^4^	1.57 × 10^4^	7.04 × 10^3^	5.76 × 10^2^
RIME	7.96 × 10^8^	4.95 × 10^8^	2.52 × 10^5^	8.86 × 10^4^	6.13 × 10^6^	1.52 × 10^6^	1.15 × 10^5^	3.74 × 10^4^	6.74 × 10^3^	8.13 × 10^2^
SSA	1.49 × 10^11^	2.84 × 10^10^	3.55 × 10^10^	5.03 × 10^9^	6.97 × 10^7^	2.67 × 10^7^	1.71 × 10^10^	1.83 × 10^9^	2.16 × 10^4^	2.48 × 10^3^
HPO	1.33 × 10^10^	9.62 × 10^9^	8.54 × 10^8^	6.34 × 10^8^	6.00 × 10^6^	6.90 × 10^6^	1.44 × 10^8^	3.22 × 10^8^	7.39 × 10^3^	7.64 × 10^2^
MSRIME	**3.87 × 10^8^**	**2.09 × 10^8^**	**6.60 × 10^4^**	**1.12 × 10^4^**	**2.52 × 10^6^**	**1.05 × 10^6^**	**5.04 × 10^4^**	**1.60 × 10^4^**	**6.04 × 10^3^**	8.22 × 10^2^
	**F17**		**F18**		**F19**		**F20**		**F21**	
	**AVG**	**STD**	**AVG**	**STD**	**AVG**	**STD**	**AVG**	**STD**	**AVG**	**STD**
PSO	5.28 × 10^3^	2.85 × 10^2^	3.20 × 10^6^	1.18 × 10^6^	5.70 × 10^6^	1.73 × 10^6^	1.73 × 10^7^	1.11 × 10^6^	3.74 × 10^3^	1.61 × 10^2^
GSA	6.32 × 10^6^	4.13 × 10^6^	4.75 × 10^7^	1.21 × 10^7^	5.75 × 10^7^	2.30 × 10^7^	1.53 × 10^10^	2.63 × 10^9^	5.43 × 10^3^	2.41 × 10^2^
GWO	5.06 × 10^3^	6.57 × 10^2^	1.38 × 10^7^	9.51 × 10^6^	9.81 × 10^6^	4.36 × 10^6^	5.13 × 10^8^	5.22 × 10^8^	3.06 × 10^3^	7.94 × 10
WOA	1.23 × 10^4^	4.23 × 10^3^	1.22 × 10^7^	4.61 × 10^6^	9.93 × 10^6^	3.34 × 10^6^	2.01 × 10^8^	8.17 × 10^7^	4.30 × 10^3^	1.29 × 10^2^
DBO	9.06 × 10^3^	1.21 × 10^3^	1.50 × 10^7^	7.99 × 10^6^	2.44 × 10^7^	1.33 × 10^7^	3.85 × 10^7^	3.68 × 10^7^	3.90 × 10^3^	2.24 × 10^2^
AVOA	6.37 × 10^3^	5.72 × 10^2^	3.51 × 10^6^	1.91 × 10^6^	**3.70 × 10^6^**	1.95 × 10^6^	1.89 × 10^6^	**4.88 × 10^5^**	3.57 × 10^3^	1.94 × 10^2^
RIME	5.57 × 10^3^	7.73 × 10^2^	7.40 × 10^6^	3.75 × 10^6^	8.69 × 10^6^	3.92 × 10^6^	1.11 × 10^7^	1.01 × 10^7^	2.89 × 10^3^	6.66 × 10
SSA	2.25 × 10^6^	6.71 × 10^5^	1.53 × 10^8^	9.98 × 10^7^	1.43 × 10^8^	4.86 × 10^7^	1.69 × 10^10^	1.83 × 10^9^	4.69 × 10^3^	1.98 × 10^2^
HPO	6.82 × 10^3^	3.04 × 10^2^	8.69 × 10^6^	1.06 × 10^7^	5.35 × 10^6^	7.85 × 10^6^	3.39 × 10^7^	5.08 × 10^7^	3.78 × 10^3^	2.77 × 10^2^
MSRIME	**4.88 × 10^3^**	**2.41 × 10^2^**	**2.66 × 10^6^**	**1.18 × 10^6^**	4.52 × 10^6^	**1.01 × 10^6^**	**1.81 × 10^6^**	3.70 × 10^6^	**2.81 × 10^3^**	**3.26 × 10**
	**F22**		**F23**		**F24**		**F25**		**F26**	
	**AVG**	**STD**	**AVG**	**STD**	**AVG**	**STD**	**AVG**	**STD**	**AVG**	**STD**
PSO	2.22 × 10^4^	5.98 × 10^2^	5.16 × 10^3^	2.89 × 10^2^	5.53 × 10^3^	3.38 × 10^2^	3.44 × 10^3^	**3.41 × 10**	2.11 × 10^4^	6.47 × 10^3^
GSA	2.49 × 10^4^	9.79 × 10^2^	8.61 × 10^3^	5.05 × 10^2^	1.30 × 10^4^	1.11 × 10^3^	2.07 × 10^4^	1.13 × 10^3^	4.58 × 10^4^	2.24 × 10^3^
GWO	2.40 × 10^4^	6.71 × 10^3^	3.63 × 10^3^	7.57 × 10	4.22 × 10^3^	1.24 × 10^2^	5.94 × 10^3^	5.24 × 10^2^	1.54 × 10^4^	**9.71 × 10^2^**
WOA	3.07 × 10^4^	2.01 × 10^3^	5.22 × 10^3^	1.99 × 10^2^	6.33 × 10^3^	3.54 × 10^2^	8.66 × 10^3^	8.52 × 10^2^	3.81 × 10^4^	2.81 × 10^3^
DBO	2.54 × 10^4^	2.90 × 10^3^	4.53 × 10^3^	1.99 × 10^2^	5.59 × 10^3^	1.80 × 10^2^	1.11 × 10^4^	8.11 × 10^3^	2.55 × 10^4^	2.01 × 10^3^
AVOA	2.00 × 10^4^	1.20 × 10^3^	4.22 × 10^3^	2.55 × 10^2^	5.16 × 10^3^	3.59 × 10^2^	4.12 × 10^3^	1.38 × 10^2^	2.53 × 10^4^	2.67 × 10^3^
RIME	2.00 × 10^4^	2.22 × 10^3^	3.54 × 10^3^	**6.89 × 10**	4.10 × 10^3^	2.26 × 10^2^	3.56 × 10^3^	6.73 × 10	1.31 × 10^4^	1.36 × 10^3^
SSA	3.51 × 10^4^	4.85 × 10^2^	7.25 × 10^3^	6.06 × 10^2^	1.20 × 10^4^	3.89 × 10^2^	2.61 × 10^4^	3.30 × 10^3^	5.01 × 10^4^	4.52 × 10^3^
HPO	2.13 × 10^4^	2.54 × 10^3^	4.22 × 10^3^	1.36 × 10^2^	5.05 × 10^3^	1.99 × 10^2^	5.00 × 10^3^	1.48 × 10^3^	2.38 × 10^4^	3.48 × 10^3^
MSRIME	**1.79 × 10^4^**	**4.60 × 10^2^**	**3.38 × 10^3^**	1.50 × 10^2^	**3.92 × 10^3^**	**1.24 × 10^2^**	**3.40 × 10^3^**	5.22 × 10	**1.30 × 10^4^**	1.42 × 10^3^
	**F27**		**F28**		**F29**		**F30**			
	**AVG**	**STD**	**AVG**	**STD**	**AVG**	**STD**	**AVG**	**STD**		
PSO	3.66 × 10^3^	2.14 × 10^2^	3.50 × 10^3^	6.62 × 10	8.99 × 10^3^	**4.68 × 10^2^**	**2.09 × 10^7^**	9.02 × 10^6^		
GSA	1.60 × 10^4^	1.66 × 10^3^	2.77 × 10^4^	1.82 × 10^3^	2.83 × 10^5^	6.67 × 10^4^	2.99 × 10^10^	2.99 × 10^9^		
GWO	4.10 × 10^3^	2.02 × 10^2^	8.08 × 10^3^	9.17 × 10^2^	**8.35 × 10^3^**	7.65 × 10^2^	4.26 × 10^8^	7.56 × 10^7^		
WOA	5.86 × 10^3^	5.94 × 10^2^	1.19 × 10^4^	1.74 × 10^2^	1.95 × 10^4^	3.84 × 10^3^	1.76 × 10^9^	8.44 × 10^8^		
DBO	4.22 × 10^3^	3.25 × 10^2^	2.07 × 10^4^	2.84 × 10^3^	1.25 × 10^4^	2.63 × 10^3^	1.47 × 10^8^	6.61 × 10^7^		
AVOA	4.33 × 10^3^	2.97 × 10^2^	4.75 × 10^3^	6.24 × 10^2^	9.56 × 10^3^	7.68 × 10^2^	2.79 × 10^7^	**8.59 × 10^6^**		
RIME	3.85 × 10^3^	1.24 × 10^2^	3.68 × 10^3^	8.13 × 10	8.87 × 10^3^	8.73 × 10^2^	1.03 × 10^8^	5.72 × 10^7^		
SSA	1.27 × 10^4^	1.22 × 10^3^	3.14 × 10^4^	2.84 × 10^3^	1.81 × 10^5^	6.64 × 10^4^	2.89 × 10^10^	5.92 × 10^9^		
HPO	3.92 × 10^3^	1.38 × 10^2^	1.34 × 10^4^	5.72 × 10^3^	9.03 × 10^3^	9.70 × 10^2^	3.95 × 10^8^	8.74 × 10^8^		
MSRIME	**3.63 × 10^3^**	**1.22 × 10^2^**	**3.46 × 10^3^**	**2.64 × 10**	8.50 × 10^3^	1.17 × 10^3^	4.73 × 10^7^	2.56 × 10^7^		

**Table 9:** CEC2022 Test Results.

	**f1**		**f2**		**f3**		**f4**	
	**AVG**	**STD**	**AVG**	**STD**	**AVG**	**STD**	**AVG**	**STD**
SOGWO	1.26 × 10^4^	3.08 × 10^3^	5.03 × 10^2^	4.27 × 10	6.05 × 10^2^	2.04	8.60 × 10^2^	2.21 × 10
IRIME	4.48 × 10^2^	7.57 × 10^1^	4.32 × 10^2^	2.91 × 10	6.03 × 10^2^	1.35	8.52 × 10^2^	1.34 × 10
EWOA	2.42 × 10^4^	9.81 × 10^3^	5.69 × 10^2^	3.58 × 10	6.62 × 10^2^	1.53 × 10	9.20 × 10^2^	3.12 × 10
IGWO	1.05 × 10^3^	6.97 × 10^2^	4.53 × 10^2^	2.46 × 10	**6.02 × 10^2^**	1.51	8.90 × 10^2^	3.79 × 10
MSWOA	2.56 × 10^4^	3.68 × 10^3^	5.73 × 10^2^	8.13 × 10	6.70 × 10^2^	1.01 × 10	9.23 × 10^2^	1.91 × 10
ACGRIME	7.18 × 10^3^	2.75 × 10^3^	4.49 × 10^2^	1.53 × 10	6.02 × 10^2^	**7.03 × 10^−1^**	8.46 × 10^2^	1.12 × 10
MISSA	1.19 × 10^4^	4.33 × 10^3^	6.04 × 10^2^	6.61 × 10	6.02 × 10^2^	6.43	9.41 × 10^2^	1.67 × 10
MSRIME	**3.00 × 10^2^**	**2.30 × 10^−2^**	**4.17 × 10^2^**	**4.15 × 10**	6.02 × 10^2^	1.47	**8.38 × 10^2^**	**1.10 × 10**
	**f5**		**f6**		**f7**		**f8**	
	**AVG**	**STD**	**AVG**	**STD**	**AVG**	**STD**	**AVG**	**STD**
SOGWO	1.01 × 10^3^	6.82 × 10	3.33 × 10^5^	6.49 × 10^5^	2.05 × 10^3^	**6.44**	2.28 × 10^3^	6.93 × 10
IRIME	1.12 × 10^3^	1.40 × 10^2^	8.04 × 10^3^	5.03 × 10^3^	2.06 × 10^3^	1.79 × 10	2.23 × 10^3^	**1.54**
EWOA	4.25 × 10^3^	2.90 × 10^3^	7.38 × 10^5^	1.01 × 10^6^	2.23 × 10^3^	2.36 × 10	2.28 × 10^3^	5.87 × 10
IGWO	1.06 × 10^3^	1.51 × 10^2^	1.75 × 10^5^	4.71 × 10^5^	2.04 × 10^3^	1.59 × 10	2.24 × 10^3^	6.78
MSWOA	4.66 × 10^3^	2.99 × 10^3^	1.01 × 10^6^	1.13 × 10^6^	2.26 × 10^3^	6.90 × 10	2.27 × 10^3^	4.78 × 10
ACGRIME	1.02 × 10^3^	1.45 × 10^2^	**3.98 × 10^3^**	**1.52 × 10^3^**	2.05 × 10^3^	1.33 × 10	2.23 × 10^3^	2.13
MISSA	2.59 × 10^3^	1.06 × 10^3^	1.58 × 10^7^	7.79 × 10^6^	2.20 × 10^3^	4.78 × 10	2.30 × 10^3^	6.52 × 10
MSRIME	**9.28 × 10^2^**	**2.51 × 10**	5.53 × 10^3^	3.86 × 10^3^	**2.05 × 10^3^**	1.67 × 10	**2.23 × 10^3^**	2.55
	**f9**		**f10**		**f11**		**f12**	
	**AVG**	**STD**	**AVG**	**STD**	**AVG**	**STD**	**AVG**	**STD**
SOGWO	2.51 × 10^3^	1.85 × 10	3.51 × 10^3^	8.24 × 10^2^	3.21 × 10^3^	3.63 × 10^2^	2.96 × 10^3^	1.62 × 10
IRIME	2.47 × 10^3^	6.47 × 10^−1^	2.67 × 10^3^	2.02 × 10^2^	3.09 × 10^3^	2.18 × 10^2^	2.90 × 10^3^	1.21 × 10
EWOA	2.56 × 10^3^	4.36 × 10	4.74 × 10^3^	1.25 × 10^3^	3.79 × 10^3^	4.56 × 10^2^	3.07 × 10^3^	8.38 × 10
IGWO	2.48 × 10^3^	1.04 × 10	3.40 × 10^3^	1.29 × 10^3^	3.15 × 10^3^	1.75 × 10^2^	2.92 × 10^3^	6.35
MSWOA	2.58 × 10^3^	4.29 × 10	4.54 × 10^3^	1.35 × 10^3^	3.44 × 10^3^	1.34 × 10^2^	3.08 × 10^3^	9.11 × 10
ACGRIME	2.48 × 10^3^	3.52 × 10^−1^	**2.51 × 10^3^**	**6.24 × 10**	2.93 × 10^3^	6.46 × 10	2.94 × 10^3^	4.05
MISSA	2.55 × 10^3^	5.00 × 10	4.80 × 10^3^	1.94 × 10^3^	3.48 × 10^3^	1.68 × 10^2^	2.96 × 10^3^	1.00 × 10
MSRIME	**2.47 × 10^3^**	**2.11 × 10^−1^**	2.61 × 10^3^	1.29 × 10^2^	**2.90 × 10^3^**	**2.27 × 10^−2^**	**2.90 × 10^3^**	**3.77**

**Table 10:** Wilcokerson rank-sum test and Friedman rank.

CEC 2017			CEC 2022		
Algorithm	+/−/=	Rank	Algorithm	+/−/=	Rank
PSO	22/3/4	4	SOGWO	11/0/1	5
GSA	29/0/0	10	IRIME	10/0/2	3
GWO	25/1/3	5	EWOA	12/0/0	7
WOA	28/1/0	8	IGWO	10/1/1	4
DBO	29/0/0	7	MSWOA	12/0/0	8
AVOA	27/1/1	6	ACGRIME	8/2/2	2
RIME	24/1/4	3	MISSA	11/0/1	6
SSA	29/0/0	9	MSRIME		1
HPO	24/1/4	2			
MSRIME		1			

**Table 11:** Experimental Results and Statistical Analysis.

Experiment Number	Algorithm	*L*	*z*	∆*avg*	∆*r*
(a)	RIME	30.2066	15	13.0125	6.0611
	MSRIME	29.6722	17	13.8139	3.4018
(b)	RIME	31.6208	16	14.7911	5.3895
	MSRIME	30.2066	10	13.8566	4.9674
(c)	RIME	31.6208	14	14.2281	6.4446
	MSRIME	29.7990	6	12.5052	5.0993
(d)	RIME	30.0285	10	13.6695	5.9711
	MSRIME	30.2066	10	13.0108	4.8837
(e)	RIME	30.2072	10	13.9165	5.0276
	MSRIME	30.2066	9	13.6783	4.2919
(f)	RIME	29.9755	7	14.2045	6.7476
	MSRIME	29.8543	12	14.6267	6.7497

**Table 12:** BCR distribution on different maps.

	Small Ordinary Map	Small Urbanization Map	Large Ordinary Map	Large Urbanization Map
Number of grid units	400	400	1600	1600
BCR	21%	35%	26%	40%
Number of building units	84	140	416	640

**Table 13:** Statistical Table of Small Map Experiment Results.

Environment	Algorithm	*L_best_*	*L_avg_*	*Time*	*z*
Small ordinary map	IRIME	28.5891	29.2471	0.7627	11
	ISSA	28.1262	29.0219	0.7639	9
	GWO	30.2858	32.6208	0.4463	10
	SOGWO	28.8514	29.0788	0.5136	9
	RIME	28.8438	30.4493	0.7429	10
	DBO	30.0512	31.964	0.6271	12
	FSA	30.4841	33.8836	0.8452	13
	MSRIME	28.0642	28.6825	0.5085	8
Small urbanization map	IRIME	29.0568	30.0427	0.7882	12
	ISSA	29.5046	30.0285	0.6379	9
	GWO	31.5246	35.3782	0.5379	16
	SOGWO	29.9482	30.6143	0.8680	9
	RIME	29.9526	32.2066	0.5122	15
	DBO	31.7514	34.3137	0.7555	13
	FSA	30.0214	32.1421	0.6680	9
	MSRIME	29.0543	30.5013	0.5610	10

**Table 14:** Statistical Table of Large Map Experiment Results.

Environment	Algorithm	*L_best_*	*L_avg_*	*Time*	*z*
Large ordinary map	IRIME	64.0248	69.8665	1.0738	7
	ISSA	66.4523	74.5615	1.8894	10
	GWO	76.8542	82.7469	0.8977	25
	SOGWO	68.4652	70.1946	1.9477	13
	RIME	65.5216	68.3745	0.8188	23
	DBO	72.3542	76.8284	0.5634	5
	FSA	70.2540	72.1567	0.9866	9
	MSRIME	62.9851	64.8854	0.8392	17
Large urbanization map	IRIME	68.2548	70.7117	1.6892	31
	ISSA	65.9546	67.1904	2.5597	39
	GWO	79.3562	86.3523	0.9827	36
	SOGWO	68.2546	72.6826	1.2707	36
	RIME	69.2548	76.8061	0.9801	36
	DBO	74.3269	77.0711	1.1861	8
	FSA	71.2398	79.8995	1.0878	11
	MSRIME	65.9856	67.0903	0.9878	26

**Table 15:** Additional test data.

Environment	Algorithm	*L_best_*	*L_avg_*	*Time*	*z*
Scenario 1	IRIME	53.1828	54.7851	1.0235	20
	ISSA	53.1828	54.2864	1.2546	20
	GWO	53.7686	55.0248	1.2350	21
	SOGWO	53.2434	54.4347	1.3425	20
	RIME	53.2118	54.4347	0.8457	21
	DBO	53.2434	55.0873	1.2482	20
	FSA	54.8537	55.6936	0.8854	20
	MSRIME	53.1828	54.1048	0.8649	20
Scenario 2	IRIME	34.3716	35.2546	0.9756	16
	ISSA	34.2587	35.7297	0.8294	14
	GWO	34.5498	35.2587	0.7102	16
	SOGWO	34.5498	35.5428	0.8529	15
	RIME	34.5498	35.7213	0.5700	15
	DBO	35.6007	39.8995	0.6661	14
	FSA	34.3716	35.8124	0.6816	15
	MSRIME	34.2587	35.5498	0.6115	14
Scenario 3	IRIME	52.4108	53.6840	1.8164	38
	ISSA	51.7108	53.5428	2.5268	37
	GWO	53.9009	55.6236	2.0216	44
	SOGWO	53.0357	55.3242	4.2314	40
	RIME	52.9961	54.2687	1.3954	33
	DBO	53.8885	54.6873	2.2486	36
	FSA	52.0321	54.9851	2.3548	38
	MSRIME	50.8190	53.0924	1.5584	33
Scenario 4	IRIME	69.3028	72.8136	1.1905	14
	ISSA	69.2983	72.0354	1.8142	14
	GWO	70.2546	79.5139	1.6541	26
	SOGWO	69.2983	74.3716	1.8546	14
	RIME	67.2779	72.6218	0.9570	23
	DBO	70.2549	76.2426	1.0144	7
	FSA	69.8423	73.4646	1.1480	7
	MSRIME	67.9775	71.2524	1.1331	11

## Data Availability

The data that support the findings of this study are available from the corresponding author upon request. There are no restrictions on data availability.
